# Emergent conservation conflicts in the Galapagos Islands: Human-giant tortoise interactions in the rural area of Santa Cruz Island

**DOI:** 10.1371/journal.pone.0202268

**Published:** 2018-09-12

**Authors:** Francisco Benitez-Capistros, Giorgia Camperio, Jean Hugé, Farid Dahdouh-Guebas, Nico Koedam

**Affiliations:** 1 Central University of Ecuador, Biomedicine Research Institute (INBIOMED), Quito, Ecuador; 2 Vrije Universiteit Brussel, Department of Biology, Laboratory of Plant Biology and Nature Management (APNA), Ecology & Biodiversity, Brussels, Belgium; 3 Université Libre de Bruxelles, Department of Organism Biology, Laboratory of Systems Ecology and Resource Management (SERM), Brussels, Belgium; 4 ETH Zürich, Department of Earth Sciences, Zürich, Switzerland; 5 Eawag, Department of Surface Waters Research & Management, Dübendorf, Switzerland; 6 University of Hasselt, Centre for Environmental Science, Hasselt, Belgium; 7 University of Ghent, Centre for Sustainable Development, Ghent, Belgium; Universitat Trier, GERMANY

## Abstract

The conservation of biodiverse areas around the world has contributed to the protection and recovery of endangered species. This has been the case for 11 species of Galapagos giant tortoises (*Chelonoidi*s spp.) that today are successfully maintained over six islands: Española, Santiago, Pinzon, Isabela, San Cristobal and Santa Cruz. A favourable state of conservation will depend however on future development in the islands. In Santa Cruz Island the development of the agricultural areas has encroached on the migratory routes of the southwestern species *C*. *porteri and* may be an emergent conflict for tortoise conservation. We investigated the social and ecological inter-linkages using two methods framed under a participatory rural appraisal (PRA) approach: semi-structured interviews and questionnaires to study farmers’ perceptions and attitudinal factors regarding giant tortoises; as well as the associated socio-economic impacts of the conflict. Moreover, we coupled the PRA approach with an ecological assessment of giant tortoises’ population density by performing transect counts during the two yearly phases of giant tortoises’ migration to the lowlands (January to June) and back to the highlands (July to December). Our results indicate that farmers reporting damage and cultivating crops have higher odds of taking actions (fencing and physical actions) towards giant tortoises; regardless of having (or not) a negative perception towards the species. The economic losses for crops and fences averaged 2.8 USD/m^2^ and 13USD/m, respectively, and provide an initial step to further analyse and characterise the direct and indirect damage costs. Finally, we estimated a density of 76 and 185 individuals of giant tortoises per km^2^ in the rural area for the lowland and highland migratory phases, respectively. Our approach provides grounded scientific social and ecological information to effectively inform and aid managers, policy and decision makers in the selection of adequate social and ecological criteria to implement the best available options in the resolution of this emergent conservation conflict.

## Introduction

Conservation challenges require new approaches to science and management. Social-ecological system (SES) research intends to integrate human and natural systems into one comprehensive system that can provide adequate and transmittable information to managers, policy and decision makers, and citizens [1–3]. Currently, SES studies are an important part of conservation research that aim at including cultural and societal structures and institutions to develop sustainable and resilient interactions between humans and nature [4]. A SES approach allows the identification of complex inter-linkages between social and ecological variables. These interactions can lead to conflict if not addressed correctly [5, 6], such as when protected wildlife raids farmers’ crops or when fencing and illegal hunting affect wildlife survival [7, 8]. Although conflicts between humans and wildlife have been widely framed as human-wildlife conflict, the term is misleading as it suggests that species are conscious human antagonists [9, 10]. These situations are better framed as conservation conflicts which are defined as: “situations that occur when two or more parties with strongly held opinions clash over conservation objectives and when one party is perceived to assert its interests at the expense of another” [11]. This means that conservation conflicts require transdisciplinary approaches from the natural, social and humanity sciences [11] and this is why an SES approach is suited to understand and contribute to solve conservation conflicts.

Important progress in conservation has allowed legitimising and protecting biodiverse areas around the world [12]. However, Protected Areas (PAs) and their surroundings (rural or urban areas) are often the stage of conservation conflicts because the crucial habitats of the protected wildlife are too restrictive, forcing wildlife to incur into human territory where development or activities such as housing and agriculture are being developed or are expanding [13–15]. The effectiveness of PAs is being contested by its “fixed” or “static” structure in the landscape that does not comply with non-static conservation targets (e.g. migratory or nomadic species) of both marine and terrestrial ecosystems [14, 16]. Moreover, PAs have often led to an exclusionary ‘nature by itself’ approach to conservation [4], where local communities have often been removed from their land with no consultation or adequate compensation, increasing conflicts between park managers and local communities which ultimately undermine conservation strategies [17].

The Galapagos archipelago is a renowned site of global conservation importance (UNESCO World Heritage site since 1972). Although often perceived as a unique and pristine natural environment, and a living museum and showcase of evolution, it is also a site where social-ecological dynamics take place and strongly interact with conservation agendas, something that is not well studied [18]. The archipelago was only formally colonized by the Ecuadorian State in 1832 and a little more than a century later, in 1959, the National Park was established following an exclusionary ‘nature by itself’ approach to conservation, allowing human settlements on 4 islands: Isabela, Santa Cruz, San Cristobal and Floreana, which represent 3% of the total islands territory (7880 km^2^). In the same year, in 1959, a status review revealed that Galapagos giant tortoises (*Chelonoidis* spp.) were at the brink of extinction after centuries of depredation by humans. First by pirates, seal hunters and whalers (17^th^ -19^th^ century), then by the excessive hunting (late-19^th^ and early-20^th^ century) and lastly by introduced rats (20^th^ century) eating tortoises’ eggs [19]. Despite the extinction of 4 of the 15 recognized species [20, 21], efforts to recovering giant tortoise populations have been particularly successful over the past 50 years. Through local captivity-breeding and restoration programmes [22, 23] 11 of the 15 different species are currently conserved in 6 islands: Española, Santiago, Pinzon, Isabela, San Cristobal and Santa Cruz. In terms of conservation conflicts, the fact that 8 of the 11 species do not have direct contact with human activities eases somewhat the conservation of giant tortoises advantageous [24].

**Fig 1:**
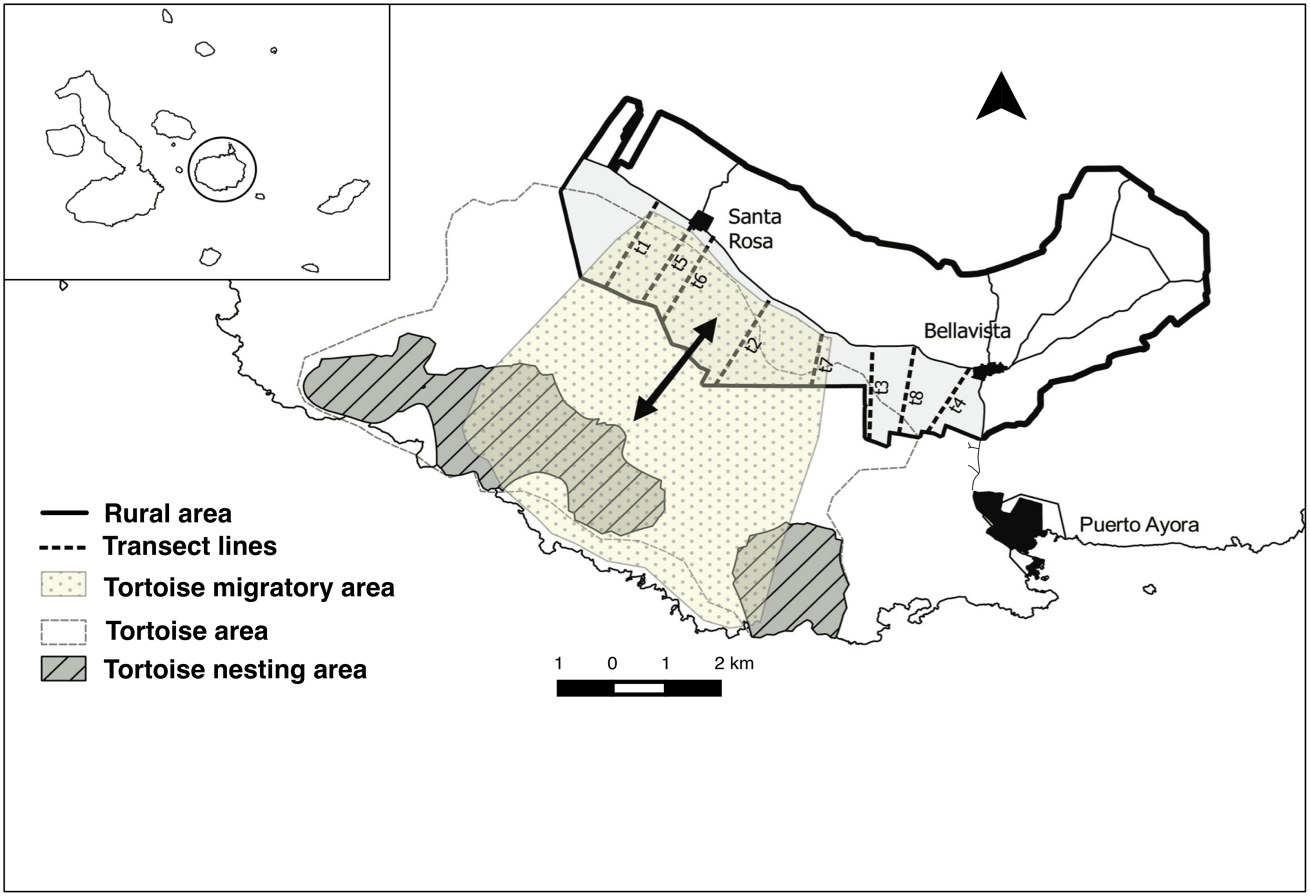
Study site area in Santa Cruz Island. The transect lines of this study are indicated with dotted lines (….) and correspond to the two transect phases. First transect phase (t1, t2, t3, t4) and second transect phase (t1, t2, t3, t5, t6, t7, t8). Santa Cruz shape file and rural area is reprinted from the Census of Agricultural Production Units under a CC BY license, with permission from the Consejo de Gobierno del Régimen Especial de Galapagos (CGREG), original copyright 2014.

In this study we focus on *Chelonoidis porteri* to illustrate an important emergent conservation conflict in Santa Cruz Island, Galapagos. With an estimated total population of 3000 individuals [25], Santa Cruz’ giant tortoises are divided by farmlands into two species: *C*. *porteri* in the southwest and *C*. *donfaustoi* in the southeast of the island. Both species with domed carapaces morphology, however differing in size and shape [21, 26]. As the only mega-herbivore to thrive in Galapagos, giant tortoises’ ecological role through seed dispersal, trampling and nutrient cycling is key for ecosystem functioning [26, 27]. However, human settlement has encroached on their migratory routes (Fig 1) where agriculture, farming and locally based ecotourism have been developed [27, 28]. Agriculture and farming are among the most disruptive human activities to biological diversity [29] and together with urbanisation, are recognised as important threats especially to the endemic biodiversity of insular ecosystems. These ecosystems are highly vulnerable and have to cope with rapid land use changes and short-term planning of human activities [30].

Although the lack of data for some islands and the coarse nature of existing maps have hampered efforts to quantify changes in vegetation in the archipelago [31], it is estimated that 50% of the total rural agricultural area of Santa Cruz Island (14842 ha) has experienced significant land use change from 1986 to 2006 [32]. Similarly, Watson et al. [33] estimated in 2010 that 25% (3 121 ha), 88% (8 381ha) and 76% (1 765 ha) of the transition, humid and very humid vegetation zones respectively, had been modified by human impacts in Santa Cruz [33]. These vegetation changes correspond to the reduction of the island’s endemic plant species such as *Scalesia pedunculata* and *Cyathea weatherbyana* and the propagation of invasive agricultural species such as guayava *Psidium guajava*, blackberry *Rubus niveus*, and cascarilla (quina) *Cinchona pubescens* [33–36]. The increasing land abandonment from farmers attracted by better economic opportunities in other sectors such as tourism, has allowed the proliferation of these invasive species in the rural areas, in all four inhabited islands [37]. Although conservation policies protect giant tortoises in Galapagos, there are no studies on the interactions occurring when giant tortoises migrate from the protected areas to the rural areas. Preventive conservation management strategies are lacking and hence understanding the factors associated with this conflict and its social-ecological inter-linkages is urgent.

Our goal is to understand, characterise and map this conservation conflict by investigating its social and ecological inter-linkages. Inspired by similar studies on conservation conflicts [38–41] we used participatory rural appraisal (PRA) as an overarching methodological approach to study farmers’ perceptions and attitudes towards giant tortoises; as well as the associated socio-economic impacts of the conflict (social factors). Furthermore, we coupled the PRA approach with an ecological assessment of giant tortoises’ population density by using field surveys during the two yearly phases of giant tortoises’ migration from and to the national park and the rural area (ecological factors). Effectively managing conservation conflicts requires a first initial mapping stage, where the identification of key stakeholders, understanding their values, attitudes, goals and positions as well as the collection of socio-economic and ecological information is necessary [11]. In this paper the in-depth analyses of social and ecological information have allowed us to initially map the conflict in place. Our results show the existence of a low negative perception about giant tortoises in our sample population of farmers (37%, N = 102). Conversely, attitudinal factors shows that regardless of the farmers’ perception, farmers with small plots of land, producing crops and reporting damage are more likely to take actions against giant tortoises (e.g. fencing and physical actions). The quantification of average economic losses for damaged crops (2.8 USD/m^2^) and fences (13USD/m) provide critical first hand information to understand the socio-economic dimension of the conflict. Lastly, the density estimation of giant tortoises in the rural area for the lowland (76 individuals/km^2^) and highland migratory phases (185 individuals/km^2^) allowed us to confirm (or reject) whether a real problem exists or was limited to a perception. By identifying key stakeholders, their perceptions and attitudes and the socio-economic and ecological impacts of the conflict we co-generated knowledge and novel information which is an essential first step before managing conservation conflicts [11] and which can help policy, decision makers and managers to improve the social-ecological fit of conservation strategies [42].

## Material and methods

### Methodological approach

Effective conservation requires the acknowledegement of a plurality of views [43]; even more so when conflicts in conservation involve specific societal groups [44] and iconic species [24, 45]. In this study we used Participatory Rural Appraisal (PRA) as the overarching methodological approach to understand farmers’ perceptions towards giant tortoise presence in the rural area. PRA is a family of methods that focuses on attitudes and behaviour, and enables local people to share, enhance and analyse their knowledge of life and conditions. PRA helps an outsider to quickly understand a system from the point of view of local stakeholders and actors [46, 47]. Framed under the PRA approach, we selected two methods: semi-structured interviews and questionnaires, coupled to transect walks to estimate the density of giant tortoises in the rural area. Zhang and Wang [38] used a similar approach to study human-elephant interaction in China.

#### Ethics statement

None of the national Ecuadorian and local agencies in charge of approving and providing research grants (e.g. SENESCYT) and/or research permits in Galapagos (Galapagos National Park, Charles Darwin Foundation) have a specific ethics committee or institutional review board. The same also applies for the *Fédération Wallonie Bruxelles*, a Belgian institution which reviewed and approved the research and provided the travel grant in 2015. However, the written approval for the use of the PRA approach and its methods (semi-structured interviews and questionnaires) was granted by the Directorate of Galapagos National Park with the research permit No: PC-39-15. Moreover, the quality and procedures of the research have been followed by the doctoral support committee at both the *Vrije Universiteit Brussel* (VUB) and the *Université Libre de Bruxelles* (ULB).

In this study a verbal consent regarding the use of the survey data was obtained from all the participants. This verbal consent consisted of informing and explaining the purpose of the surveys, respecting the anonymity of all interviewees and questionnaire respondents, the identity of the surveyor and the researchers’ stated objective to disseminate subsequent research results.

### Data collection

**Fig 2:**
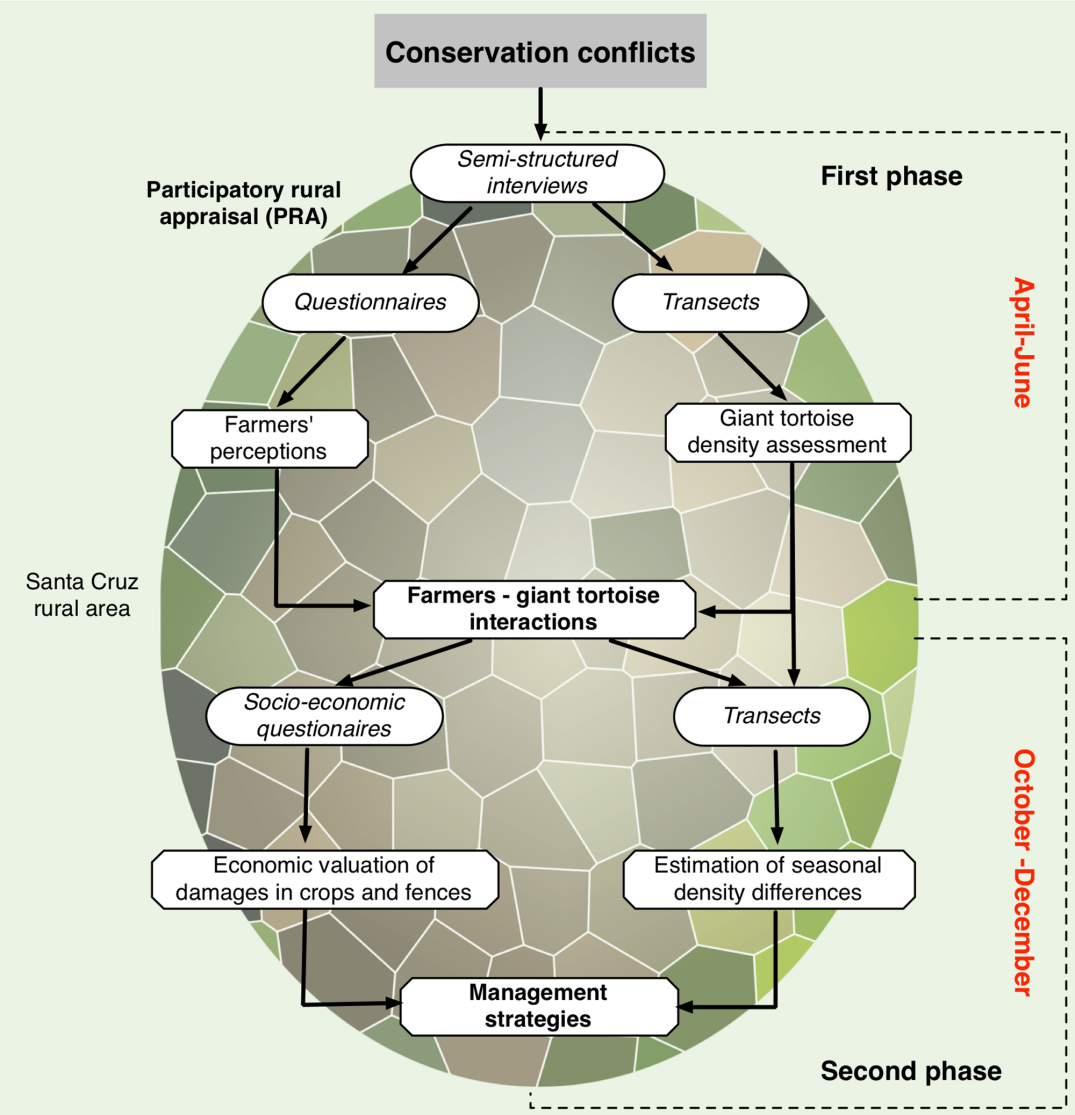
Research methodological framework during the two research phases. Italics indicate the method used to collect data.

We collected data in two phases (Fig 2). In the first one (April to June 2015) we conducted semi-structured interviews (n = 18) and subsequently built a questionnaire to gather information on the perceptions and attitudinal factors of farmers (n = 102) (from here on referred to as first phase questionnaire). In the second phase (October to December 2015) we built a socio-economic questionnaire to quantify damage caused by giant tortoises to as experienced by landowners (n = 53). Both times, we used the transect method (Fig 1) to estimate the population density of *C*. *porteri* during the corresponding migratory seasons [21, 27].

#### Semi-structured interviews (SSI)

SSI rely on a preconceived interview guide with standard questions which are asked in each separate interview, allowing comparison and maintaining data quality [48]. Compared to structured or unstructured interviews, SSI have a more flexible design that allows questions to be followed up with comments, prompts and additional relevant questions that may develop during the interview [49]. This flexibility is key for complex conservation issues such as conservation conflicts [50] and studies on conservation science-policy interfaces [48]. Moreover, SSI are most appropriate to design a set of precise questions that would be needed for a survey or questionnaire [49, 51] which was one of the aims of this research. Furthermore, in this research we systematized the semi-structured interviews [52] so that each open question would allow us to retrieve factual information about: the study area, farmer’s perceptions and attitudes towards the farmer-tortoise interaction [53]. The 18 semi-structured interviews were conducted between April and May 2015 (S1 Table).

All interviewed persons were informed about the purpose of the research. We obtained the identity of the interviewer and their agreement regarding the use of the information we obtained. All interviews were conducted in Spanish, the language spoken by all the interviewees and the interviewers’ mother tongue (co-first authors of this paper). The interviews were recorded when allowed by the speakers; otherwise the interviews were manually transcribed on paper. Of the eighteen interviews, five were identified through a list of contacts provided by the Ministry of Agriculture (MAG), six were interviewed during informal encounters, one was approached at the local market, two were approached through snowballing (a sampling technique that follows recommendations from other interviewees of the study), two were contacts established through previous research [24, 54]. In addition, we interviewed three non-farmers: two employees of the MAG, both responsible for the agricultural sector of the island, of whom one was previously involved in a tortoise monitoring programme with the Charles Darwin Foundation and the National Park; and one employee of the National Park, involved in giant tortoises’ management and monitoring. We conducted eleven of the interviews in the farmers’ premises. During these visits we collected information regarding the areas and logistics, which proved useful in the elaboration of the transects and the questionnaires.

#### Questionnaires

In this research, questionnaires were used: 1) to gather quantitative data on farmers’ perception on the presence of giant tortoises in the rural area; and 2) to retrieve baseline socio-economic information about damage caused by giant tortoises.

#### First phase questionnaire

With the information gathered through the semi-structured interviews, we built a close-ended and multiple-choice format questionnaire to gain baseline quantitative information. In total 102 questionnaires were fully completed between May and June 2015, which corresponds to 29% of the total 357 censed farmers in Santa Cruz [55]. We approached the respondents whose farms were located in different zones of the rural areas in order to have an adequate sample of the area by covering a maximum of diversity, in terms of farm characteristics. The first seven completed questionnaires were used as a pilot test, to formulate the definitive version of the questionnaire by clarifications and improvements regarding the understanding of the interviewer’s purpose. These were not included in further analysis. Questionnaires were conducted at the local market, while visiting the farms door to door, at recreational sites, outside churches, at local restaurants and on public transport. The questionnaire was divided into three main sections: 1) Questions to collect demographic information, 2) questions related to the characteristics of the farm type, and; 3) questions related to the farmers’ perception on tortoises (S2 Table). All respondents had to be minimum eighteen years old, and knowledgeable about farm activities (owners, workers or relatives of these). As indicated earlier, all questionnaire respondents were informed about the purpose of the survey, the identity of the surveyor and the planned use of the information we obtained and hence agreed with the use of the survey data.

#### Second phase: Socio-economic questionnaires

Based on the methodologies used in conservation conflicts (e.g. involving elephants [56]) we collected field data by means of a socio-economic questionnaire (S3 Table). The results of the first phase questionnaire allowed us to make a distinction between ‘non-damage presence’ and ‘presence causing damage’ by the wildlife, which was derived by the occurrence of giant tortoises and the actual damage to crops and fences. Henceforth, our second phase socio-economic questionnaire was built to determine the presence of giant tortoises and occurrence of damage to crops and fences per month, season, and area (km^2^). Note that the word ‘damage’ is used to describe the disturbance interaction from wildlife to humans (e.g. to crops and fences). However, in the other direction of the interactions (humans to wildlife) we call the disturbance interaction: habitat loss, fragmentation or fencing.

We considered the area of damage (m^2^) and frequency (years) as the baseline parameters for measuring crop damage [57]. However, in order to have a more reliable estimation of the respondents reporting [58, 59] we included three parameters to assess the crop damage: 1) Type of damage in 6 categories (1 = <5%, 2 = 6–10%, 3 = 11–20%, 4 = 21–50%, 5 = 51–80%, 6 = >80%), 2) crop quality, (good/medium/poor); and, 3) crop stage (seeding/intermediate/mature) (S3 Table). Additionally, we included a section for the estimation of the damage to fences (highly reported in farmers with damage in the first phase questionnaire) including the height of the fences on their lands; and a final open question to investigate local perspectives on alternatives to avoid damage by giant tortoises (S3–V Table).

**Fig 3:**
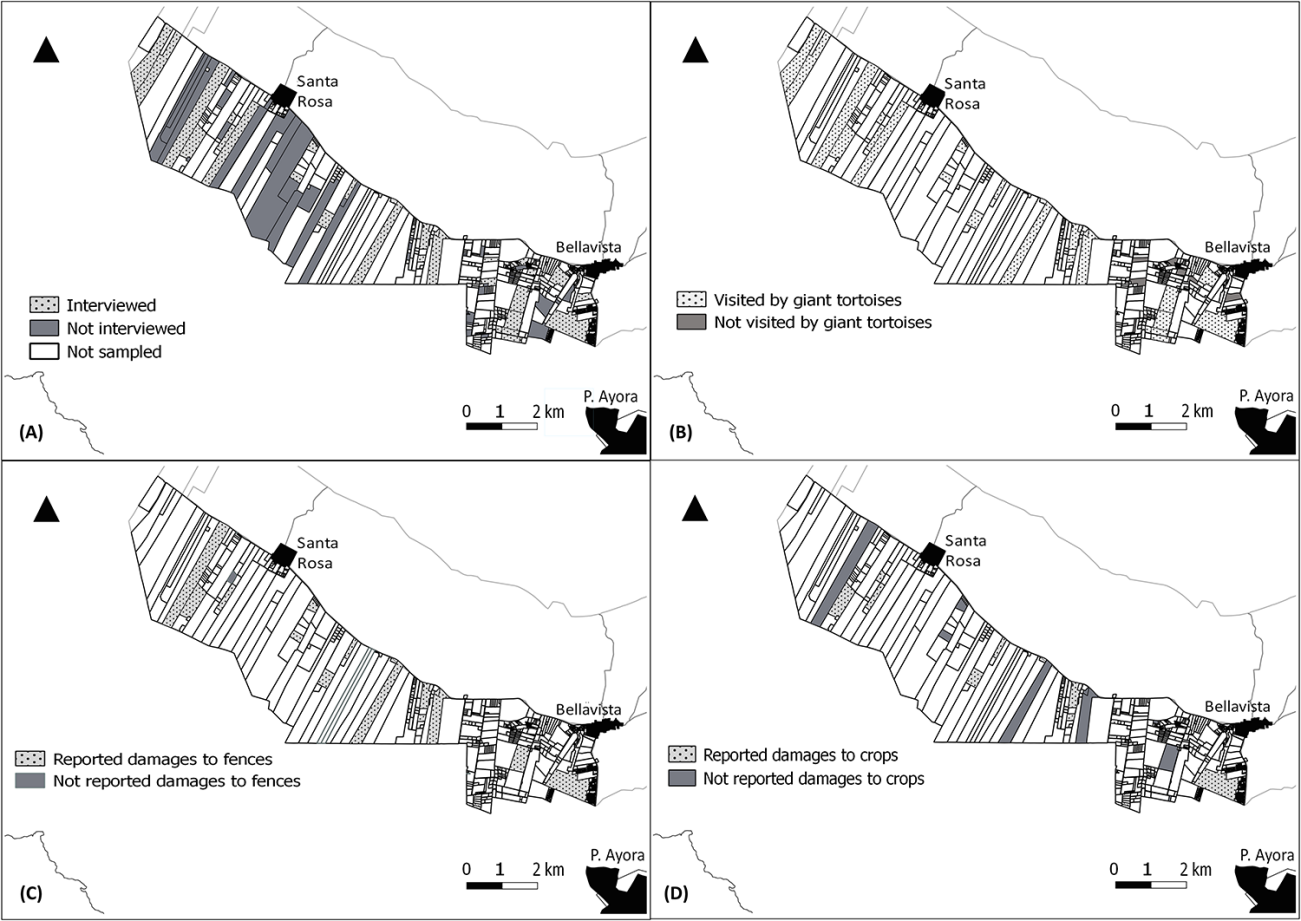
Stratified random sample of study area for socio-economic questionnaires. (A) Completed questionnaires (n = 53). (B) Landowners reporting giant tortoises entering farms (n = 41). (C) Landowners reporting damages to fences (n = 16). (D) Landowners reporting damages to crops (n = 9). Santa Cruz shape file and rural area plots of land is reprinted from the Census of Agricultural Production Units under a CC BY license, with permission from the Consejo de Gobierno del Régimen Especial de Galapagos (CGREG), origin al copyright 2014.

To collect these data we first identified the areas where giant tortoises were present, based on the results of the first phase questionnaire where farmers had reported damage in the southwestern section of the rural area (Fig 1). Secondly, with the Geographic Information System (GIS) land maps produced in 2015 provided by the Galapagos Governing Council (GGC), we selected landowners independently of their registered activity (i.e. agriculture, timber, and conservation) from the latest census [55], as we identified inconsistencies in this census through preliminary questionnaire-based controls. For example, some of the landowners registered as practicing agriculture had abandoned lands, a common occurrence in Galapagos since invasive plants (i.e. *Rubus niveus*) then proliferate rapidly [34, 35]. We randomly sampled 25% (N = 95) of the total 384 registered landowners in the southwest area, in order to have a representative sample (Fig 3). In total we retrieved 53 fully completed socio-economic questionnaires. The remainder of 42 landowners (44%) could not be sampled because they were not in Galapagos, had abandoned lands, had passed away, and/or were not willing to participate.

#### Density estimation of giant tortoises in the rural area

We outlined and defined transects to evaluate the relative density of tortoises in the rural area by means of distance sampling [60–62]. Estimating animal density through transects is a widely used method [28, 61, 63–65] suited for large terrestrial herbivores [66]. Leuteritz et al. [65] used this method to estimate tortoise density. On Santa Cruz island, the migration of giant tortoises occurs in two periods which are associated with the foraging behaviour and the seasonal weather patterns in the highlands: 1) a migration to the highlands from July to December; and, 2) a migration from the highlands to the lowlands in the rainy season from January to July [27]. Therefore, transect surveys were conducted during these two phases (Fig 1).

#### First transect phase (April-June 2015)

In the first set of transects we performed preliminary transects based on the information collected through the semi-structured interviews. Four transects (Fig 1) were selected based on the ease of access, the authorisation of the landowners, and the distribution in the selected study area (t1, t2, t3 and t4). Note that t4 was not included in the density estimates and not replicated in the second set of transects since it was crossing a densely human-inhabited area. Each transect was repeated three times. The first set of transects was performed during the rainy season in the lowland [27], thus we assessed tortoise density during the migration towards the lowlands. This allowed us to measure the minimum density of tortoises in the rural area, which could be considered as the period of ‘minimum potential interaction’ between tortoises and farmers. Each time a tortoise was encountered during the transect lines, we recorded the geographic coordinates of the location of the tortoise with a GPS device (GARMIN GPSMAP 62s). In this first transect phase, the perpendicular distance of the tortoise from the transect line was measured visually [67] and recorded by trained observers [60, 67].

#### Second transect phase (October–December 2015)

Following the same approach and methodology as in the first transect phase (April-June), we established seven transect lines, approximately every 2 km (Fig 1), starting from the three established transects during the first set of transects (t1, t2, t3, t5, t6, t7, t8). Each transect was repeated four times. The period corresponded to the highland rainy season when tortoises migrate to the rural area for foraging [27]. This allowed us to measure the maximum density of tortoises in the rural area, which could be considered as the period of ‘maximum potential interaction’ between tortoises and farmers. We recorded each tortoise geographic coordinates using a GPS device (GARMIN GPSMAP 62s) along each transect and transect repetition. We then measured the perpendicular distance of each encountered tortoise using a telemeter (Nikon Aculon 911). Note that the approaches followed to estimate the density were not exactly the same in the two phases. This is due to both logistical (accessibility of the transects) and methodological reasons, such as an improved transect design, more accurate supporting materials (e.g. telemeter) and expert advice from a retired CDF giant tortoise scientist and a biodiversity consultant. Although different approaches were used to measure the distance from the transect lines during the two phases (visual vs. telemeter), it is important to know that although visual estimates (used in the first set of transects) might seem less accurate, it has been shown that if the observers are trained, understand the distance sampling method and are familiar with the specie and environment, the estimation of distance is not problematic [67].

### Data analysis

#### Analysis of variables and prediction model

After analysing the first phase questionnaire we retrieved six *ex-post* variables that describe the interaction between farmers and giant tortoises: 1) Farm production, 2) farm dimension (ha), 3) farm bordering the national park or not, 4) reported damage, 5) perception, and 6) actions (S1 Dataset). The variable farm production was grouped into farms that either reported crop cultivation (or not) and those that reported cattle rearing. We made this choice because mono-productive farms are almost inexistent in the rural area of Santa Cruz, and often its labourers set a small plot of land aside, dedicated to crop cultivation for family subsistence (chacra). Most of the farms in Santa Cruz are mixed, usually presenting several concurring farming activities such as: crop cultivation, cattle rearing, coffee plantations and/or are farms dedicated to tourism. The ‘perception’ variable was retrieved from three different questions in the first phase questionnaire (Q17-Q27-Q28) as a triple check, since we considered that the farmers would not report negative perception straightforwardly (S2 Table). We considered a farmer as having a negative perception when at least one of the three questions was selected:

Q17) Multiple-choice type:
  *Which are the plagues that affect your farm*?: tortoises (N = 6)
Q27) Multiple-choice type:
  *According to you tortoises are*: a plague (N = 4), a nuisance (N = 14)
Q28) Yes/No question: type:
  *Do you consider tortoises in your farm to be a problem*?: yes (N = 22)

The ‘action’ variable consisted of indirect and direct actions taken by farmers to diminish/avoid interactions with tortoises. Indirect actions involved the use of fences, which were built to avoid tortoise entering the land. These fences had a height of ≤15 cm from the ground, five or more lines of barbed wire or fully obstructing structures (wood, rocks, walls), which halt or discourage giant tortoise or other large animals from entering (e.g. pigs, cows). Direct actions involved any physical interaction with giant tortoises to keep them out of the farm (e.g. harassing, displacing and turning tortoises downside up). All categorical variables met the assumptions for *Chi* square test (χ^2^ = *p*<0.05). The Wilcoxon-Mann-Whitney test was used to describe the central tendency between categorical variables and the continuous variable farm dimensions. For the prediction model, to explain if a farmer was willing to take actions (direct and indirect) on giant tortoises we ran a binary logistic regression to check if significant variables (χ^2^ = *p*<0.05) predicted the dependent variable “actions”. All the assumptions for the prediction model of the logistic regression were met.

#### Socio-economic valuation

Damage to crops and fences were quantified throughout the second phase socio-economic questionnaire data using descriptive statistics *(frequencies*, *average* and *maximum costs)*. Total costs estimates were provided by the respondent’s own estimation of the damages in last event/year by area (m^2^) for crops and length (m) for fences (S2 Dataset). We finally summarise the qualitative information on the reported alternatives to avoid damages by giant tortoises.

#### Giant tortoises population densities

To estimate tortoise density in the rural area we used DISTANCE 6.2 software [60, 61] to design and analyze data collected measuring the perpendicular distance of tortoises encountered along the transect lines in both research phases (S3 and S4 Datasets). According to Leuteritz et al. [65] the application of this method fits tortoise density estimates since the four main assumptions to assure the precision of the analysis are respected: i) all the objects encountered on the transect line were sighted, ii) objects do not move, iii) distance is measured or estimated with accuracy and iv) each animal observation is independent. Following Buckland et al. [60] we first analysed the data checking for outliers and applying the due truncation required in order to find the best model that could describe the animal detection function. Truncation means that we excluded all the tortoises that are detected much further (outliers) than the others individuals [60]. Then, we chose the model best fitting the detection function based on the Akaike’s Information Criterion (AIC) values and goodness-of-fit tests generated by the software DISTANCE.

## Results

### First phase questionnaire

#### Farmers and lands characteristics

**Fig 4:**
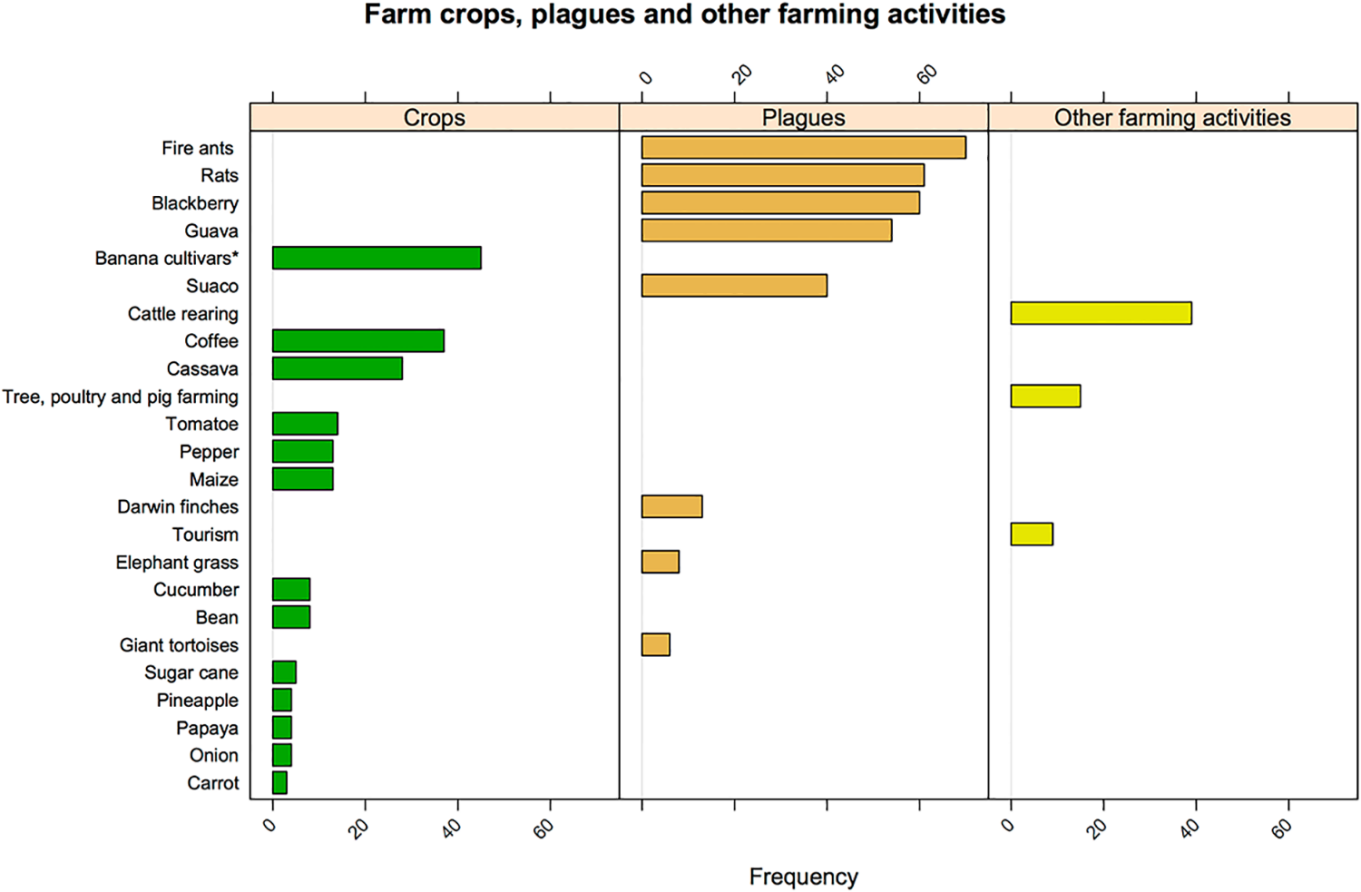
Main characteristics of the sampled farms. Animal plagues are fire ants (*Solenopsis geminata* and *Wasmannia auropunctata*) and rats (*Rattus rattus* and *Rattus norvegicus*); whereas plant plagues are blackberry (*Rubus* spp.), guayava (*Psidium guajava)*, elephant grass (*Pennisetum purpureum*) and sauco (*Cestrum auriculatum*). (*) Indicates a variety of grown banana cultivars (e.g. guinean, green bananas, plantain). Frequency refers to the absolute number of farmers reporting the respective characteritics over the total sample (*N* = 102).

Of the total 102 questionnaire respondents, only a fifth (21%, *N* = 21) did not report cultivating crops and almost half of these reported cattle rearing as the predominant activity (*n* = 10). However, the majority of farmers reported cultivating crops (79%, *N* = 81). Of these, a fourth (24%, *n* = 19) exclusively cultivated crops (short cycle crops and perennial banana cultivars), whereas the rest (61%, *n* = 62) mixed it with other activities. These other activities included cattle rearing, perennial coffee plantations, touristic farming and other activities (i.e. tree, poultry and pig farming). The most frequently reported cultivated crops, as well as plant and animal plagues are shown in Fig 4. Plagues (as characterized by the respondents) included the endemic Darwin finches (*Geospiza* spp., *Camarhynchus* spp., *Platyspiza crassirostris*, *Certhidea olivacea)*, and giant tortoises (*Chelonoidis porteri*). The farms dimensions ranged from 0.2 ha to 400ha (*median* = 21 ha, *SD* = 73.23), covering an area of 4 962.25 ha which accounts for half (53%) of the total rural area [68].

#### Analysis of variables

The presence of giant tortoises in the farms was reported by a majority of respondents (81%, *N* = 83) and was associated with cattle rearing farms (χ^2^ = 16.06, *p* < .001). More than half of the farmers (57%, *n* = 47) that reported the presence of giant tortoises reported damages, of which 95% (*N* = 42) reported damages to crops (*N* = 32) and fences (*N* = 30). The ‘damage’ variable was significantly associated with farmers that cultivated crops (χ^2^ = 3.93, *p* = .*048*). Moreover, farm dimensions of farmers that reported damages were significantly smaller (*Mdn* = 16 ha) than those that did not report damage (*Mdn* = 47.5 ha), (*U* = 526.5, *z* = -2.606, *p* = .009). A negative perception towards giant tortoises was found in 36% (*N* = 37) of the total sampled farmer population. No significant relation was found between a negative perception and the presence of giant tortoises (χ^2^ = 0.34, *p* = .558). However, a significant relation was found when considering the negative perception and the occurrence of damage, (χ^2^ = 15.88, *p* < .001). Moreover, we could determine that a negative perception was significantly associated with the median of farm dimensions (*Mdn* = 20 ha and *Mdn* = 39.5 ha, *U* =567, *z* = - 2.064, *p* = .039). More than half of the farmers that reported the presence of giant tortoises (58%, *N* = 48) took action against them. Indirect actions were the most frequent ones (48%, *N* = 23), followed by farmers that took both type of actions (33%, *N* = 16). The remainder took exclusively direct actions (19%, *N* = 9). Actions (direct and indirect) were not associated with negative perception (χ^2^ = 2.27, *p* = .132). However, both direct and indirect actions were associated with damage (χ^2^ = 18.23, *p* < .001 and χ^2^ = 6.89, *p* = .009, respectively) and with the farmers that cultivated crops (χ^2^ = 4.50, *p* = .034 and χ^2^ = 9.63, *p* = .002, respectively). Only direct actions were significantly associated with smaller farms (*Mdn* = 35.64 ha) as compared to larger farms (*Mdn* = 70.54 ha) (*U* = 457, *z* = -2.57, *p* = .010). There were 44 farms bordering the National Park (43%). When considering the presence of tortoises, this was significantly higher than in those farms that did not border with the National Park (χ^2^ = 6.12, *p* = .013). Farmers whose properties were bordering the National Park reported less damage compared to those that did not border the park (χ^2^ = 6.12, *p* = .013) and had a significantly lower negative perception (χ^2^ = 8.38, *p* = .004). Also, these farmers were taking significantly less action towards tortoises (χ^2^ = 6.45, *p* = .011). Farms bordering the National Park had significantly lower crop cultivation (χ^2^ = 6 8.63, *p* = .003) and significantly higher cattle rearing (χ^2^ = 23.58, *p* < .001) and touristic activity (χ^2^ = 6.93, *p* = .008). Farms bordering with the National Park were significantly larger (*Mdn* = 79 ha) than those that were not bordering the park (*Mdn* = 6 ha) (*U* = 2.202, *z* = 6.56, *p* < 001). A summary of the Pearson’s chi-square and the Mann-Whitney U test results is reported in Table 1.

**Table 1:** Summary of the significant (*) and non-significant (n.s.) relation between the variables.

	Tortoises	Damage	Negative perception	Actions	Border NP
**Tortoises**	\	\	n.s.	n.s.	* (+)
**Damages**	\	\	*(+)	*(+)	* (-)
**Negative perception**	n.s.	*(+)	\	n.s.	*(-)
**Actions**	\	* (+)	n.s.	\	*(-)
**Small farm dimension**	n.s.	*(+)	*(+)	*(+)	*(-)
**Production**	* CR(+)	*CC(+)	n.s.	* CC(+) * CR (-)	*CC(-)

The signs (+) and (-) indicate positive or negative significant interactions, respectively. Cattle rearing (CR), cultivated crops (CC).

#### Prediction model

The logistic regression model supported the hypothesis that increasing levels of damage, increasing negative perception and increasing intensity of production increased farmer’s actions (direct and indirect) against tortoises (χ^2^ = 26.29, *p* < .001). The model explained 41.3% (Nagelkerke *R*^*2*^) of the variance of the dependent variable “actions” and correctly classified 79.2% of cases. Positive predictive value (farmers taking actions) was 79% and negative predictive value (farmers not taking actions) was 71%. Two of the predictor variables were statistically significant: reporting damages and cultivating crops (Table 2). Both reporting damage and the cultivating crops were associated with an increased likelihood of taking actions. Farmers that cultivated crops had eight times higher odds to take an action (OR = 8.27 C.I. = [2.01–34.02]) on tortoises than the farmers that did not cultivate crops. In addition, farmers reporting damage had almost seven times higher odds to take actions (OR = 6.84 C.I. = [2.05–22.83]). The large variation in the C.I is explained by the small sample size.

**Table 2:** Results of the binomial logistic regression predicting the likelihood of taking an action on giant tortoises.

Independent variables	B	SE	Wald	Df	p	Odds Ratio	95% CI for Odds Ratio	
							Lower	Upper
**Negative perception**	-.16	.65	.06	1	.802	.848	.234	3.07
**Damage**	1.92	.615	9.78	1	.002	6.84	2.05	22.83
**Cultivated crops**	2.113	.721	8.59	1	.003	8.27	2.01	34.02
**Coffee**	-.58	.58	1.01	1	.314	.56	.18	1.74
**Cattle rearing**	-.91	.58	2.40	1	.12	.40	.13	1.27
**Tourism**	.322	.747	.185	1	.67	1.38	.32	5.97
**Constant**	-2.19	.683	10.3	1	.001	.111		

The B coefficient shows the change in the log odds (alternate way of expressing probability) for one unit change of the dependent variable. SE = standard error. The Wald test is used to determine statistical significance. Df = degrees of freedom. Odds ratio informs about the odds that the dependent variable would change for one unit change of the independent variable. Upper and lower Confident Intervals (CI) for the odds ratio.

#### Second phase questionnaire: Socio-economic valuation

**Fig 5:**
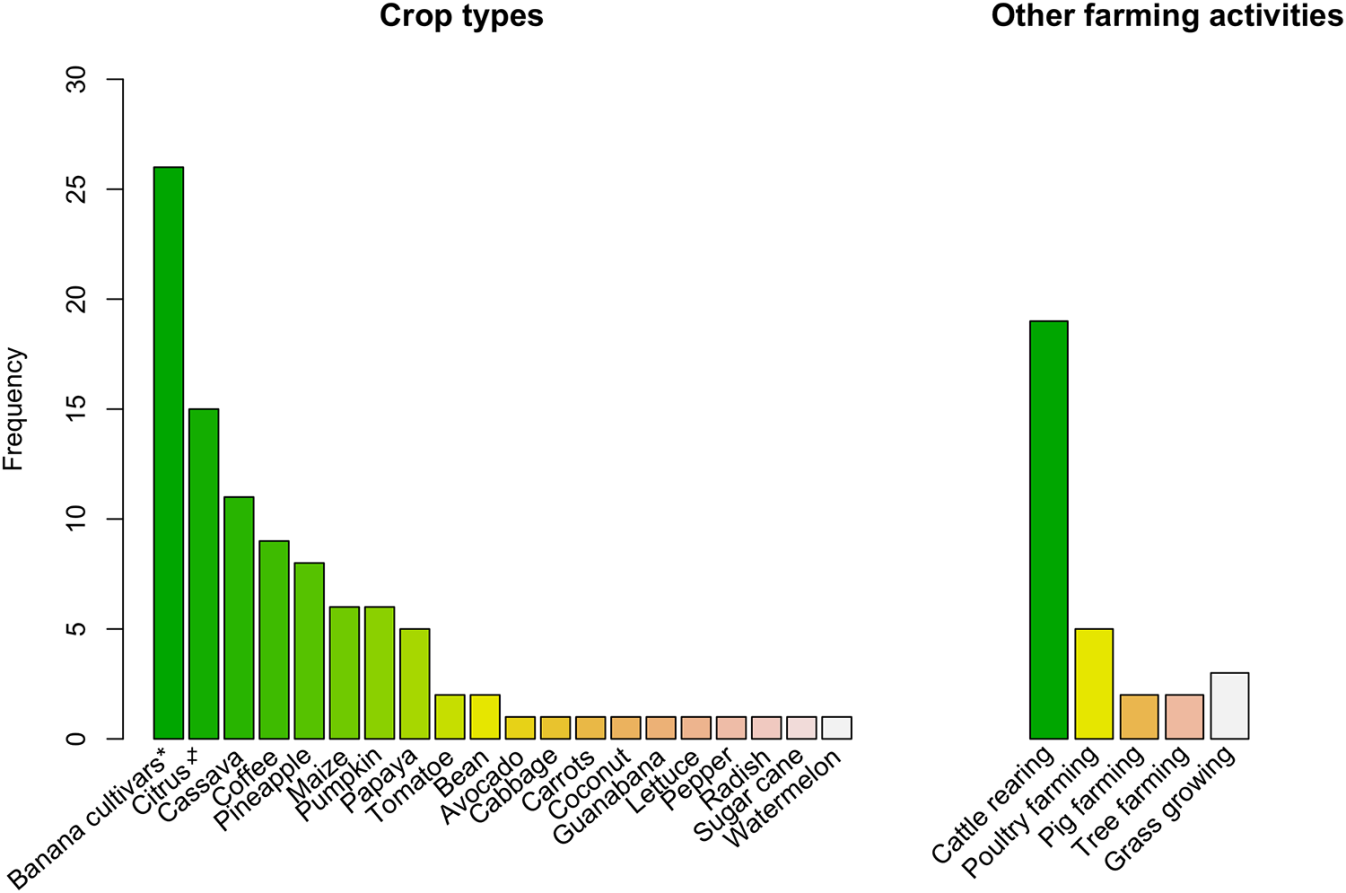
Main crop types and other farming activities as established during the socio-economic valuation questionnaire. Total sample n = 53. (*) Indicates a variety of grown banana cultivars (e.g. Guinean, green bananas, plantain) and (‡) a variety of citrus (e.g. oranges, lemons, mandarins).

Of the 53 respondents 20% (*n* = 11) reported cultivating crops as their primary economic activity, 11% (*n* = 6) livestock rearing (cattle, poultry, piggery) and the remainder 69% (*n* = 36) reported other activities (mainly services). The majority of the respondents (86%, *n* = 46) reported to have a usable productive land (crop cultivation, cattle rearing, tree farming, mixed), with many (64%, *n* = 34) also reporting cultivating crops but not as their primary economic activity. Moreover, 70% (*n* = 37) of the lands did not border the National Park. The average land size was 20.82 ha (*median = 4 ha*, *SD = 43*). The most commonly cultivated crops were banana cultivars, citrus, cassava, coffee, pineapple, maize, pumpkin and papaya (Fig 5). The total number of landowners who reported giant tortoises entering their farms was 41 (77%), with 25 (47%) reporting damage to either fences or crops. Although 30% (*n* = 16) reported damage to crops; only 17% (*n* = 9) were able to provide an estimate of the economic costs of the damaged crops during the last event/year and 30% (*n* = 16) of the fencing costs (Table 3 and Fig 5).

**Table 3:** Sample characteristics of the socio-economic valuation in the rural area of Santa Cruz Island (n = 53).

**Characteristic**	**Sub-characteristic**		**Detail**
Crop cultivation as primary economic activity			11
Crop cultivation			30
No crop cultivation			23
Crops damaged by giant tortoises	**Species**	**Frequency**	**(%)**
	a. Banana cultivars	7	23
	b. Maize	6	20
	c. Pineapple	5	17
	d. Cassava	2	7
	e. Coffee	2	7
	f. Grass for forage	2	7
	h. Watermelon	2	7
	i. Cat’s claw	1	3
	j. Vegetables	1	3
	k. Papaya	1	3
	l. Pumpkin	1	3
Total reporting crop economic losses	9		
Crops economic losses	Average: 1194 USD, Maximum 6000 USD		
Economic losses per unit area (USD/m2)*	Average: 2.80 USD, Maximum 8 USD		
Total reporting fencing costs	16		
Fencing costs to deter giant tortoises*	Average: 1974 USD, Maximum 9000 USD		
Fencing cost per meter (USD/m)*	Average: 13 USD, Maximum 125 USD		
Compensation		Non existent	
**Alternatives to prevent damages (n = 5)**			
To provide economic incentives for building touristic infrastructure in the land instead of agriculture. To promote growing crops that will not be damaged by giant tortoises such as: oranges, cocoa and teak trees for timber. To promote the local agricultural market in particular for those whose subsistence relies solely on agriculture. To design efficient anti-tortoises fences that do not cause damage to tortoises nor landowners, as well as ecological corridors. To build and set more (non damaging) fences in the side of the GNP.			

* USD currency rate corresponds to December 2015

#### Economic costs: Damaged crops and fencing

**Fig 6:**
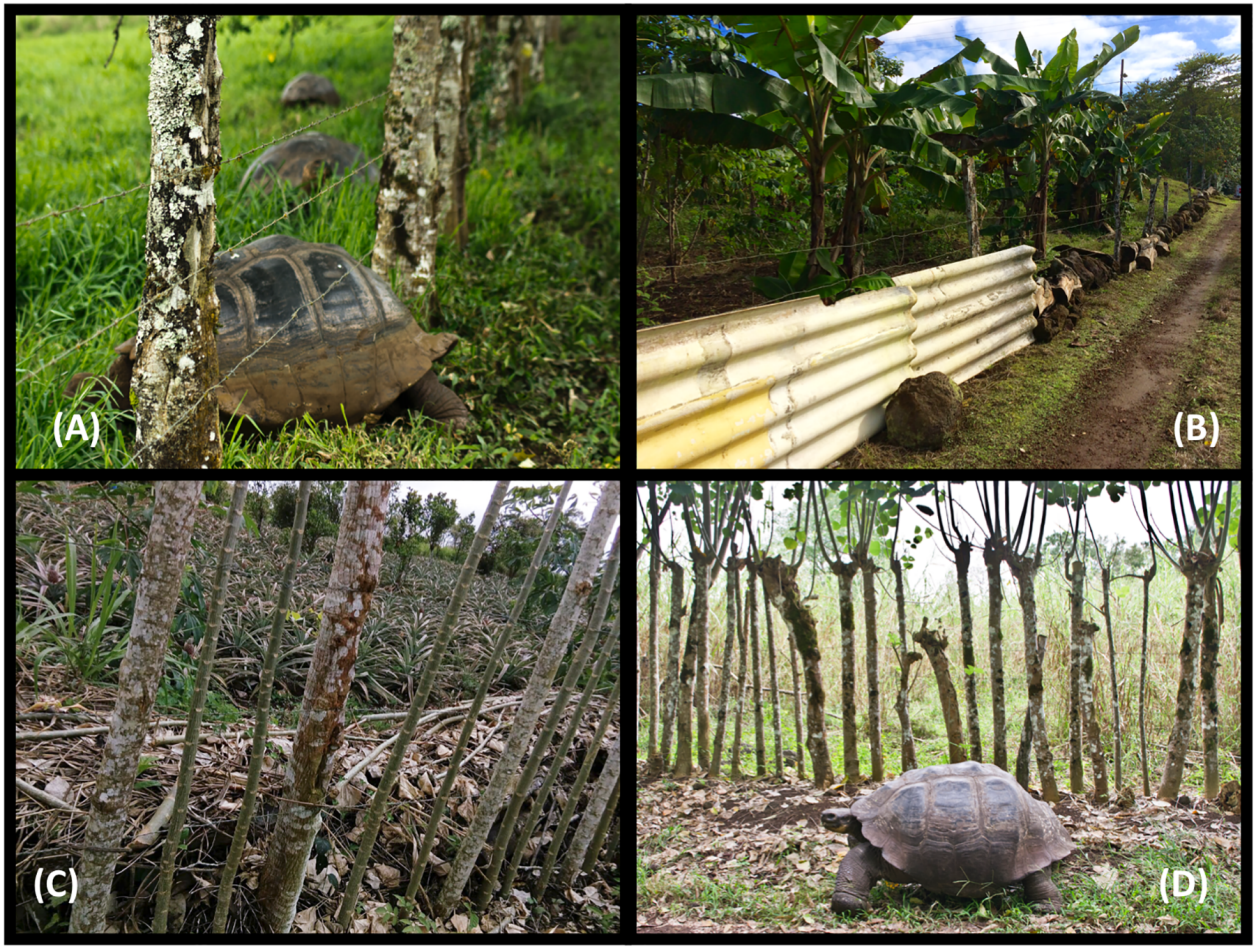
Example of fences that allow or avoid the entrance of giant tortoises in the farmlands. (A) Barbed wire fence at > 15cm from the ground allowing the entrance of giant tortoises. (B) Tin roof and rocks obstructing fence. (C) Porotillo and barbed wire obstructing fence. (D) Porotillo obstructing ‘live fence’.

Crop damage was reported from May to December; but the highest numbers of incidents (7 of 9) were reported for the months of November and December. Although our aim was to obtain the landowners report for the last year (2014–2015), this was only possible with 5 respondents. Conversely, with 2 of them we could verify the magnitude of losses (S1 Fig). The reported crop damages per area (ha) varied from <5% to >80%. The majority of the lost crops were allegedly of good quality with varied aging stages (seedling, intermediate, mature) (Appendix 3-III). As indicated in Table 3, the average reported economic loss for crop damage was 1194 USD with an average cost of 2.8 USD/m^2^ of unit land. Similarly, the fencing cost average was 1974 USD with an average cost per meter of fence of 13USD/m. Note that as shown in Fig 6, there are different types of materials used in the construction of fences such as barbed wire, rocks, brick walls and porotillo and these might explain the large variation in the reported costs per meter (Table 3).

#### Landowners’ alternatives to prevent damages

The majority of the landowners (75%, *n* = 40) reported the use of physical barriers by fencing either with barbed wire, rocks and/or porotillo as the alternative to prevent damages by giant tortoises in their croplands. Other suggestions by the respondents (*n* = 5) included: 1) to provide economic incentives for building touristic infrastructure in the land instead of agriculture; 2) to promote growing crops that will not be damaged by giant tortoises such as: oranges, cocoa and teak trees for timber; 3) to promote the local agricultural market in particular for those whose subsistence relies solely on agriculture, 4) to design efficient anti-tortoises fences that do not cause damage to tortoises nor landowners, as well as ecological corridors; and 5) to build and set more (non-damaging) fences in the side of the GNP (Table 3).

#### Density estimation of giant tortoises in the rural area

Table 4 shows the calculated density estimates for the first and second set of transects. The results show an estimated density of 76 tortoises per Km^2^ (along 50.33 km of line transects) and 185 tortoises per Km^2^ (along 79.2 km of line transects) for the first and second transect sets respectively.

**Table 4:** Estimated tortoise density for both sets of transects, expressed as number of individuals per km^2^.

Transect set	No. Individuals	No. Individuals Truncated	Effective strip width	Density of individuals	95% CI Lower Upper		CV
1	122	116^a^	13.53	75.70^c^	53.34	107.41	0.18
2	685	681^b^	22.40	184.92^d^	116.5	293.52	0.23

Effective strip width refers to the software estimated width of the transects, 95% lower and upper Confidence Interval (CI) and coefficient of variation are shown.

^a^Truncation of the outliers set at a distance of 39 m.

^b^Truncation of the outliers set at a distance of of 155 m.

^c^ Best density functions fitting the data was the hazard-rate function with the cosine series extension adjustment (S2 Fig).

^d^ Best density functions fitting the data was the hazard-rate function with the hermite polynomial adjustment (S3 Fig).

## Discussion

Our results have allowed us to retrieve information about the current status of the conservation conflict between giant tortoises and farmers in the rural area of Santa Cruz Island. Our findings show that in general, farmers in Santa Cruz do not have a negative perception about giant tortoises. Yet, regardless of the perception, farmers with small plots of land, producing crops and reporting damages to crops take more actions against the giant tortoises (e.g. fencing, displacing, harassing them). Additionally, although only perceptually, we quantified the damage costs to crops and fences which is an initial step to understand the socio-economic dimension of the conflict. Finally, using the transect method we estimated giant tortoise density in the rural area in the two migratory phases (lowland and highland), which allowed us to confirm that the emergent conservation conflict in Santa Cruz is real.

In this paper we can see that the heterogeneity of farming system makes the identification and categorisation of specific variables key to study the interaction between farmers and giant tortoises. It is important to note that, although the migratory routes of giant tortoises between the National Park and the rural area of Santa Cruz Island have been well described and studied [26, 27, 69] this research provides a first mapping step to manage this emergent conservation conflict. Having used PRA as an overarching systematic approach has proven to be effective, as it has allowed us to identify and characterise conservation conflicts related to agro-ecosystems and tortoises in Santa Cruz Island, a framework which maybe applicable to other conservation conflicts on Galapagos. In line with Chambers [46] we also consider that semi-structured interviews (SSI) formed the essential first step of PRA in this research. SSI allowed us to structure our research in three main clusters of information through questionnaires and transects: 1) farmers’ perceptions and actions, 2) socio-economic valuation of damage; and 3) population density of giant tortoises in the rural area. In this sense, we agree with Mueller et al. [70] on the advantage of using PRA to combine local knowledge inquiry with scientific study at a low cost. PRA has been an adequate tool for a preliminary assessment and mapping of conservation conflicts [71] but that still has limitations. For example, we acknowledge that the results of the quantification of damage might be biased. This bias is the result of the limited numbers of participants that could provide us with an economic estimation of crop damage (n = 9) and fences (n = 16); and to the fact that the results are only based on perceived and not verified real damage costs. A final limitation of the PRA approach is related to the integration of the ecological data with the socio-economic information. However, it is important to note that although the approaches might be complementary and help in the future management of the conflict, interpolating these data is not easy or necessarily correct.

### Farmers’ perceptions and actions

Giant tortoises were present commonly in the study area, in particular in farms with cattle rearing. Although a negative perception towards giant tortoises was not predominant in our sample population, farms with the cultivation of crops and farmers reporting damage were associated with a negative perception. Similarly, those farmers reporting damages to crops were taking more actions, particularly indirect actions (use of fences) to protect their fields. In fact, fencing and direct actions against giant tortoises were taken regardless of the negative perception. It is important to note that direct actions are probably a conservative estimate, since many actions might not have been easily admitted or reported by the interviewees. Nevertheless, the large group of farmers without a negative perception could be considered as a ‘tolerant’ group. Understanding the motivation underlying such attitudes could be fundamental in addressing strategies to avoid conservation conflicts [72]. Moreover, the tolerance could be linked to the effectiveness of the prolonged conservation awareness that the iconic giant tortoises have generated in Galapagos society [24], this is epitomised in statements like: “they were here before us”, “they are part of the landscape” often reported during the SSI. Moreover, this could also be the result to a habituation process to animal presence [73, 74], in our case as an acceptance of tortoise presence and associated effects in the area.

We also found that farmers who had small farms and farmers cultivating crops more often reported giant tortoises as a cause of damage. Naughton-Treves et al. [75] argue that smaller land plots are more prone to suffer animal incursions (e.g. from elephants) than larger farms. The perception of damage is then associated with the dimension of the land. However, note that though land size and farm activity might seem causally related (i.e. large size farms rearing cattle), external factors such as improved access to productivity enhancing institutions, technologies and inputs, might shift farms’ activities and productivity [76] in the areas where giant tortoises are seasonally established and are not negatively perceived. For instance, large cattle rearing farms could change their activities to crop cultivation and be more prone to damage, thus changing the perceptions towards the specie.

On the other hand, the larger the land, the more opportunities for diversifying productive activities (i.e. livestock, tourism); consequently generating alternative sources of income and potentially less vulnerability to the wildlife [7]. Indeed “being at risk from a threat is not necessarily the same as being vulnerable to it” [77]. For example, we expected that farmers bordering the National Park would be more vulnerable and more prone to report damage, as shown by other studies where the distance from the protected areas is a predictor of damage [7, 75, 78–80]. However, our results show that farmers and respondents active on farm for which land bordered the National Park (mostly cattle rearing and touristic farms) although they were reporting a higher number of giant tortoises also reported less damage. Therefore, the distance to the National Park was not a predictor of damage; but rather the size and type of the primary productive activity that is being developed in the farm. Most of the actions that were undertaken to prevent damage were fencing plots (indirect actions). Our prediction model allows us to confirm that farmers reporting damage and cultivating crops in their lands had higher odds of taking actions to limit entrance of giant tortoises. Although, from a farmer’s perspective tortoises are generally not perceived negatively, such perceptions are not necessarily constant through time, e.g. a season of failing crops may exacerbate negative perceptions. In effect, despite the fact that they can cause damage, farmers have found strategies to deal with tortoise incursions. These strategies can prove costly and unsustainable both for giant tortoises (disrupting their migratory routes) and for the farmers (economic costs).

### Quantification of damage

Losses to important perennial crops (banana, pineapple, coffee) and short cycle crops (maize, cassava) in Galapagos were reported. Moreover, damage was mostly reported during November and December, which covers the highland migratory phase. This can be considered as the ‘period of high interference’ between giant tortoises and farmers. The quantification of spatial and temporal aspects of crop losses is essential for managing and mitigating potential conservation conflicts [81]. Our results allow us to provide an initial measurement of the associated costs of damage to crops and fences. These results will facilitate a further analysis and characterisation of damage costs (direct and indirect) and possible compensation schemes. It is important to note that landowners were the primary source of information and although this has allowed us to reduce costs and time, the approach might lack an objective reporting/recording of information due to changes in perceptions and memory influenced by events that may take on a greater significance in retrospect [58]. To minimise this inherent bias we tried to collect information only from the latest event year (2014–2015). However, the low number of landowners reporting economic losses in our sample forced us to consider a larger time frame (since 2007). Moreover, other factors can also influence this perception of costs including for example, the fact that the minimum net wage in Galapagos (659 USD) is 1.8 times higher than on the mainland, generating a market speculation in the archipelago. Another factor for example, could also be the use of porotillo (*Erythrina fusca*) for fencing. Porotillo is an introduced common flowering tree in Galapagos which can be extracted under the authorisation of the GNP [82] and which is commonly used as cheaper alternative and long standing ‘live fence’ to prevent the entrance of giant tortoises and large animals. Accordingly, the highest average cost (125 USD/m) seems to be an over-estimation of the respondents, because the estimated construction cost in Galapagos is 552 USD/m^2^ or 23.5 USD per meter [83]. Thus, we can see that there is a mismatch of the information cost between what was reported by some participants and what might actually be the real cost. The tendency to overestimate cannot be explained on basis of the results, but it may be related to the fact that it is perceived as a net cost and not as an investment, hence carrying a negative perspective. Thus, the reported costs for damaged crops and fencing have to be interpreted with caution and should ideally be complemented with field-based monitoring in the croplands over time [56]. Nevertheless, the results of the socio-economic questionnaire allowed us to verify the increasing land abandonment [37, 54]. This situation warrants particular attention not only for the future of the intended agricultural development in Galapagos [24], but also for the implications for the migratory routes of Santa Cruz giant tortoises. Abandoned lands typically exhibit a proliferation of invasive species such as blackberry (*Rubus* spp.), which become true vegetative barriers for the passage of giant tortoises as observed during the transects and are at the expense of native flora. Moreover, in combination with the extensive sectors with low fencing (<15cm), which also block passage of giant tortoises, a reduction of the current suitable foraging and migratory ‘rural’ area for giant tortoises is to be expected. A continuous monitoring of giant tortoise population density and distribution throughout the rural area will be key for detecting and preventing decreasing population trends as well as to favour an effective conservation [84]. Ideally, the density estimates should be integrated into a complete monitoring framework that includes management and environmental covariates.

### Density estimation

Our density results allow us to provide an overview of the differences in the densities of giant tortoises in the two migratory seasons. As verified, lower and higher densities correspond to the yearly migratory seasonality of giant tortoises from the protected (national park) to the non-protected (rural) area in Santa Cruz [26, 27, 69]. Former studies in Galapagos have focused on determining the density of giant tortoise in the National Park where most conservation efforts have taken place to restore the endangered species of giant tortoises. In Santa Cruz Island the estimated density of *Chelonoidis porteri* in the National park was of 720 individuals per km^2^ [25]. In our research, we did not aim to give an absolute density estimates for giant tortoise in Galapagos but rather we aimed to identify and assess potential conflicting interactions between tortoises and humans. Our results are then restricted to density estimates for the rural area as not all tortoises migrate from the National Park to the highlands [27]. Our estimated density in the rural area for the first phase of ‘minimum potential interaction’ (April-June) is almost 10 times lower (76 individuals per km^2^) than what was previously reported [22]. Similarly, for period of high interference’ (October-December) it is almost 4 times lower (185 individuals per km^2^).‘The difference between our results and what was previously reported highlights the need for further tortoise population density assessments and the differences of methods and different environments (e.g. rural vs. protected areas) which can influence the comparisons between surveys [85, 86].

Considering that giant tortoise habitat was once extending across the rural area [19] the population of giant tortoises could have undergone an “edge effect” just outside the National Park. Fencing as a strategy of protection can threaten tortoise migratory routes impeding animals’ movement along the ecosystem gradient [13, 87]. We consider that the distance sampling method as outlined in the study (Fig 1) is well suited for establishing long-term monitoring schemes. This will allow having precise and regular information on giant tortoise abundance and trends, a critical step to ensure adequate and adaptive sustainable management schemes in conservation [88–90]. Studying and protecting wildlife–in particular migratory species–in non-protected lands is essential, as these areas play a crucial role within an ecological network and are key in maintaining connectivity between the protected areas [91, 92].

### Towards an alternative and adaptive co-management

As we have detailed, this research studied and unveiled different elements of the emergent conservation conflict in the rural area of Santa Cruz Island. Addressing social-ecological dynamics is not an easy task, and needs to include adequate parameters that can explain and transmit the -sometimes complex- information to policy and decision makers [6]. There is a need of serious future actions, which we now have proposed in a currently on-going project in Galapagos where the results of this work will be used to start a participatory process in the resolution of this emergent conservation conflict. For now the findings and analysis of this study, raise important points for addressing conservation management implications:

1. Size and type of production in the land were predictors of perceived damage by giant tortoises. A smaller agricultural (crop-producing) farm will most likely have/report damage by giant tortoises. Yet, we need to stress again that large mono-productive farms are almost inexistent in the rural area of Santa Cruz, and often its labourers set a small plot of land aside, dedicated to crop cultivation for family subsistence (chacra). This situation should be taken into account in any conflict management strategy, which would focus only on the part of the population that “seems” to be more affected (i.e. small scale agriculture). Otherwise this could lead to the risk of shifting the problem to the neighbours [77] and/or shifting the problem to those actors with less (bargaining) power (labourers vs. landowners).
2. Co-generation of knowledge with the people involved in the conflict is essential to meet conservation objectives [93]. In this research we aimed at including local knowledge with the use of the PRA approach. For example, the suggested alternatives provided in this study for mitigating conflicts (Table 3) are a necessary step towards inclusive sustainable adaptive wildlife management [88, 94]. However, some of these require further evaluation in order to prevent similar mistakes that have occurred in other non-Galapagos settings with wildlife management. The following conflict mitigation strategies warrant/deserve particular attention:
  - Fencing with barbed wire: it could represent a serious issue for giant tortoises. Not only because barbed wire fences can represent a pitfall for tortoises as it is for other animals attempting to cross them [95, 96] but also because it is very likely that an effective ever-widening network of fences following a future (and very likely) land subdivision can have a detrimental effect on wildlife migratory species [e.g. 97] by reducing their foraging range and movement patterns. Indeed, interfering with migratory connectivity through human land use (change) can be one of the major threats to species conservation [13].
  - Community-based wildlife tourism (CBWT): can be traced as one of the main ideas prevailing in the community-based conservation discourse in Galapagos [24]. In the present study CBWT was also frequently reported (directly or indirectly) through the interviews and questionnaires as an appealing alternative for many landowners and farmers to diversify their activities and revenues. Policies to favour CBWT can raise tolerance and prevent retaliations to wildlife by the empowered communities because wildlife is valued as property [98]. However, valuing a species as a product (e.g. natural capital) in a society with low conservation awareness and that is mainly driven by economical pursuit could have detrimental ecological effects [24]. Likewise, inequalities in the distribution of CBWT benefits are also an issue, in particular in poor, rural communities [99]. Problems arise when governmental or non-governmental market-driven approaches to conservation favour certain consumers and producers, or worse, when local communities are excluded from the main profit of wildlife tourism as well as access to crucial natural resources [100, 101]. There are several factors for the success of CBWT (see [100, 102]), but three could be highlighted as most relevant: First, the initiative of CBWT needs to be originating from the local communities and not by external organisations with particular economic interests. Second, local communities need realistic information on the available options to develop touristic infrastructure. This information could be provided be an external organisation or independent sources but with no personal stake or narrow interests. Third, local communities need to build capacities to improve their skills to understand the real access to capital and market in place. This will allow local communities to understand what are the real costs and benefits of CBWT. Therefore, the decisions on venturing in the enterprise of CBWT will not be filled with un-realistic communal expectations such as that the communal benefits of tourism will offset the cost of living with wildlife. Such benefits need to be understood as social (e.g. communal projects for schools, clinics) rather than financial (e.g. net individual economic benefits) [99, 100].
3. Finally, as we have discussed along in this paper, the implications of the intended agricultural development in Galapagos, with particular emphasis in Santa Cruz rural area, needs to include specific and reliable social-ecological information for an accurate understanding of the dynamics in place. Here we emphasise the human-giant tortoises’ interface, but an ecosystem wide approach must eventually be included because a good management of giant tortoises only may not be sufficient to guarantee the adequate management of other endemic and native species. The temporal information on the population status of giant tortoises together with the spatial and quantitative information on crop damages and fencing costs, allows us to provide initial study elements to start addressing this “emergent” conservation conflict in Galapagos. However, the quantification of damage to crops and fences is limited by the number of participants and by the fact that they are only based on perceived and not verified real damage costs. In the future, it will be necessary to further collect more detailed and verified information regarding the real damage costs of crops and fences. Moreover, in the case that compensations or subsidies for fences are applied, then the impact on the fragmentation and disruption of giant tortoises migratory connectivity will be absolutely needed.

## Conclusions

The dynamics of linked human and ecological systems require predictive research that is able to inform interventions to conserve biodiversity while sustaining human livelihoods [103]. Our study provides grounded scientific (ecological) as well as community-based information (social), which we consider essential as a first step for a proper co-management of conservation conflicts. Moreover, we emphasise two negative issues and three positive actions that need attention before any conservation/development intervention:

### Negative issues requiring action

- Abandoned lands and the proliferation of invasive plant species such as blackberry (*Rubus* spp.) and guayava (*Psidium guajava*). Combined with the giant tortoises’ role as long distance seed dispersers on the island, this proliferation of invasive plants has recently been shown, under future climatic change scenarios, to be a potential threat to local plant communities in the low arid areas of the national park [104].
- The unregulated physical barriers used to protect crops (in particular with barbed wire) and subdivision of lands.

### Positive actions

- Field-based monitoring of croplands and further characterisation of damage costs (direct and indirect) and possible compensation schemes.
- Systematic (at least yearly) monitoring scheme of giant tortoise abundance and trends in the rural area.
- An inclusive participative decision-making process for conservation conflict resolution.

## Supporting information

S1 Table Demographic elements of the interviewees in the SSI and contacted means. (PDF)

S2 Table First phase questionnaire. (PDF)

S3 Table Second phase: Socio-economic questionnaire. (PDF)

S1 Fig Documented damaged crops. a) Farmer 1 reporting possible entrance of giant tortoises to his farmland; b) giant tortoise in corn crop field; c) damaged corn at an intermediate stage; d) farmer 2 showing damages by giant tortoises to pineapple crops; e) mature pineapple crop eaten by giant tortoises. Photographs by F. Benitez-Capistros, November, 2015.
(TIFF)

S2 Fig First set of transects. Distance detection function truncated at 39 meter, fitted with the hazard-rate function with a cosine series extension adjustment (model chosen based on the comparison of the AIC values).
(TIFF)

S3 Fig Second set of transects. Distance detection function truncated at 155 meter, fitted with the hazard-rate function coupled with the hermite polynomial adjustment (model chosen based on the comparison of the AIC values).
(TIFF)

S1 Dataset First phase questionnaire. (XLSX)

S2 Dataset Second phase socio-economic. (XLSX)

S3 Dataset First set of transects. (XLSX)

S4 Dataset Second set of transects. (XLSX)
