# Supplementary material for: Emergent conservation conflicts in the Galapagos Islands: Human-giant tortoise interactions in the rural area of Santa Cruz Island
Source: PLoS One. 2018 Sep 12;13(9):e0202268. doi: 10.1371/journal.pone.0202268 (PMC6135374; doi:10.1371/journal.pone.0202268)
Supplement: S1 Table — (PDF) [file pone.0202268.s001.pdf]

S1 Table. Demographic elements of the interviewees in the SSI and contacted means

No. Interviewee	Activity	Farm visited	Sex	Contacted through:
1.	Crop cultivation	yes	M	MAG
2.	Crop cultivation	yes	M	Market
3.	Coffee plantation and crop cultivation	No	M	Snowball
4.	Small agriculture and cattle rearing	Yes	F	Snowball
5.	Crop cultivation and cattle rearing	No	M	Casual encounter
6.	Coffee plantation	Yes	F	MAG
7.	Cattle rearing	Yes	F	Casual encounter
8.	Cattle rearing	Yes	M	Casual encounter
9.	Cattle rearing	No	M	CGG
10.	Cattle rearing	Yes	M	Casual encounter
11.	Tourism	Yes	F	Previous research
12.	Tourism and cattle rearing	Yes	M	Previous research
13.	Tourism	Yes	M	Casual encounter
14.	Tourism, crop cultivation and cattle rearing	Yes	M	MAG
15.	Crop cultivation and cattle rearing	No		MAG
16.	Ministry of Agriculture	-	F	MAG
17.	Ministry of Agriculture	-	F	MAG
18.	Galapagos National Park	-	M	-GNP
