# Supplementary material for: Emergent conservation conflicts in the Galapagos Islands: Human-giant tortoise interactions in the rural area of Santa Cruz Island
Source: PLoS One. 2018 Sep 12;13(9):e0202268. doi: 10.1371/journal.pone.0202268 (PMC6135374; doi:10.1371/journal.pone.0202268)
Supplement: S2 Table — (PDF) [file pone.0202268.s002.pdf]

S2 Table First phase questionnaire

Questionnaire number___________________ **Date: / /201**

Sex: M ☐ **F☐**

1. **¿En qué área se encuentra el terreno?** *(In which area of the map is your farm located?)*

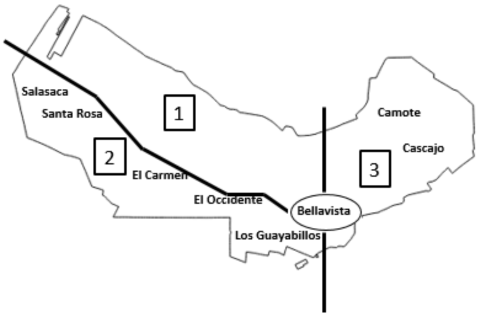

☐ 1
☐ 2
☐ 3
☐ Otro_______________________________

2. **¿El terreno limita con el Parque National?** *(Does the farm border the National Park?)*
☐ SI ☐ NO

3. **Usted es:** *(What is your role in the farm? Worker, owner, other)*
☐ Trabajador
☐ Propietario (pariente de propietario____)
☐ Otro_______________________________

4. **¿Usted vive en el terreno?** *(Do you live in the farm?)*
☐ SI ☐ NO

5. **En qué año llego a Santa Cruz?** *(When did you arrive in Santa Cruz?)*
☐ Menos de un ano
☐ Entre 1 y 10 anos
☐ Más de 10 anos
☐ Vivo acá desde siempre

6. **¿Cuántas hectáreas tiene el terreno?** *(Which is the extension of the farm (ha)?)*
☐ Entre 0.1 y 5
☐ Entre 5 y 20
☐ Más de 20
☐ No se

7. **¿Hace cuanto tiene o trabaja en el terreno?** *(Since when you work/own the land of the*

farm?)
☐ Menos de un ano
☐ 1-10
☐ Más de 10 anos

8. **¿A qué grupo de edad pertenece? En que año nació?** *(To which age class do you belong? Year of birth?)*
☐ 18-25
☐ 26-50
☐ más de 50

9. **¿A qué se dedica primariamente en su terreno?** *(Which is the main activity conducted in the farm? Sign more than one if necessary)*
☐Ganadería
(Ha____)
☐Agricultura
(Ha____)
☐Café
(Ha____)
☐Madera
(Ha____)
☐Turismo
(Ha____)
☐Nada
☐ Otro______________________________

10. **¿Qué tipo de cerca utiliza para limitar el terreno?** *(Which of the following types of fence do you use to limit your farm?)*
☐ Una, algunas o todas estas técnicas: Porotillo sembrado a menos de 30 cm uno del otro, por lo menos 5 filas de alambre de púa a menos de 5 cm de la tierra, tablas de madera, tablas de madera más alambre púa, rejas, cercos de peregrina.
(Porque?____________________________________)
☐Alambre de púa a una altura de mínimo 50 cm del suelo
☐Nada
☐ Otro______________________________

11. **Se dedica a la agricultura en una parte del terreno?** *(Do you have agriculture in a portion of the farm?)*
☐ SI
(Ha____________________Que:____)
☐ No

12. **¿Qué tipo de cerca utiliza para limitar esta parte de terreno?** *(Which type of fence do you use to limit agriculture, (if you use one)?)*
☐ No tengo agricultura

☐ Una, algunas o todas estas técnicas: Porotillo sembrado a menos de 30 cm uno del otro, por lo menos 5 filas de alambre de púa a menos de 5 cm de la tierra, tablas de madera, tablas de madera más alambre púa, rejas, cercos de peregrina.
(Porque?____________________________________)
☐Alambre de púa a una altura de mínimo 50 cm del suelo
☐Nada
☐ Otro______________________________

13. **¿Tiene cafetal?** *(Do you have coffee plantations)*
☐ SI (Ha____________________)
☐ NO

14. **¿Qué tipo de cerca utiliza para limitar esta parte de terreno?** *(Which type of fence do you use to limit coffee plantations, (if you use one)?)*
☐ No tengo cafetal
☐ Una, algunas o todas estas técnicas: Porotillo sembrado a menos de 30 cm uno del otro, por lo menos 5 filas de alambre de púa a menos de 5 cm de la tierra, tablas de madera, tablas de madera más alambre púa, rejas, cercos de peregrina.
(Porque?____________________________________)
☐Alambre de púa a una altura de mínimo 50 cm del suelo
☐Nada
☐ Otro______________________________

15. **¿Tiene invernaderos***? (Do you have greenhouses)*
☐ SI (Cuantos?___________________________________De que?_________________________)
☐ NO

16. **¿Tiene pozas?** *(Do you have water ponds?)*
☐Artificial permanente (Cuantas?___________________________)
☐Natural permanente (Cuantas?_____________________________)
☐ Natural efímera (Cuantas?______________________________)
☐No tengo pozas

17. **¿Cuál considera ser la plaga que afecta su producción y como la elimina?** *(Which one you consider as a pestilence in your farm? How do you get rid of them?)*
☐Hormigas____
☐Guayaba____
☐Rata____
☐Mora____
☐Tortuga____
☐Sauco____
☐Pinzón____
☐Pasto elefante____

☐Otro____

18. **¿Qué planea hacer con la finca en los próximos 10 años?** *(Which are your plans for the far in the next 10 years?)*
☐Seguir en lo mismo
☐Turismo
☐Cultivo
☐Ganadería
☐Vender
☐Comprar más tierra
☐Parcelar/lotizar (con lotes menos de 10 ha)
☐Edificar
☐Patrimonio familiar
☐No se
☐ Otro________________________________

19. **¿Alguna vez vio tortugas en el terreno?** *(Have you ever seen tortoises in the farm?)*
☐SI
☐NO

20. **¿Cuando fue la última vez que vio tortuga en el terreno?** *(When was the last time you saw a tortoise in the farm?)*
☐ Nunca he visto tortugas

21. **¿Cuales el número máximo de tortugas que entraron en su terreno en un día?** *(Which is the maximum number of tortoises that entered in the farm in one day?)*
☐0
☐Menos de 10
☐Entre 10 y 50
☐Más de 50

22. **¿Cuál es el número mínimo de tortugas que entraron en su terreno en un día?** *(Which is the minimum number of tortoises that entered in the farm in one day?)*
☐0
☐Menos de 10
☐Entre 10 y 50
☐Más de 50

23. **Usted en el terreno ha notado**: *(Did you notice any change in the number of tortoises in the farm?)*
☐Un aumento de las tortugas____
☐Una disminución____
☐Ningún cambio____

☐Nunca he visto tortugas en el terreno____
☐No se

24. **¿Que hacen las tortugas cuando entran en el terreno?** *(What do tortoises do when they get in the farm?)*
☐Pasan, comen y destruyen (Que?_________________ Cuál es el número máximo de tortugas que puede/podria soportar en su terreno?____)
☐Pasan, comen pero no destruyen
☐No entran (Porque____)
☐No se
☐Otro_____________________________

25. **Usted, cuando las tortugas entran en el terreno:** *(Which actions towards tortoises do you undertake when they are in the farm?)*
☐Les viran
☐Las echan
☐Las desplazan
☐Las dejan
☐Llaman al Parque Nacional/otra institución(____)
☐Les dan comida/agua
☐La admiran
☐No entran tortugas
☐ No se
☐Otro_____________________________

26. **Que usted sepa cuando las tortugas entran en los terrenos, que hacen las otras personas?** *(Do you know which actions towards tortoises are undertaken by other farmers when tortoises get in their farm?)*
☐Les viran
☐Las echan
☐Les dan comida/agua
☐Las desplazan
☐Las comen
☐Las matan
☐Llaman al Parque Nacional/ otra institución(____)
☐ Las dejan
☐La admiran
☐No se
☐Otro_____________________________

27. **Las tortugas en el área rural son***: (Tortoises in the rural area are:)*
☐Una plaga

☐Una molestia
☐Unas mascotas
☐Parte del paisaje
☐Un recurso económico
☐Las representantes de nuestra identidad
☐Las dueñas de la isla
☐No se
☐Otro____________________________

28. Usted considera las tortugas en el área rural un problema? *(Do you consider tortoises as an issue when in the rural area?*
☐ Si
☐ No
☐ No se

29. Quiere tener tortugas en su terreno? *(Would you like to have tortoises visiting your farm?)*
☐SI
(Cuantas?__________________________Porque____________________________________)
☐NO
☐No se

30. Las tortugas tendrían que estar: *(Where do you think tortoises are supposed to stay?)*
☐En el parque nacional
☐En corrales
☐En el área rural
☐En las fincas turística
☐Por toda la isla
☐No se
☐Otro____________________________

31. Alguna vez se fue a ver tortugas de visita o turismo? *(Have you ever been to see tortoises in a touristic farm or in a park pen? If yes, where and when?)*
☐ SI (Cuando la ultima vez?___________________________En donde?_______________________)
☐ NO

32. A quien acude si necesita suporte con la presencia de tortugas en su terreno? *(Who do you ask for support if you find tortoises in your farm?)*
☐Nunca tuve tortugas en el terreno
☐Instituciones públicas locales (☐PNG ☐juntas parroquiales ☐municipios ☐Otro_________________)
☐Instituciones gubernamentales (☐MAG ☐Ministerio de turismo ☐Ministerio del Ambiente ☐Otro________________)
☐Organizaciones no-gubernamentales (☐FCD ☐WWF ☐Conservation Internacional ☐Otro_______)
☐Personas de la comunidad/amigos
☐No acudo a nadie

☐No necesito suporte
☐Otros____

33. **Quien se tiene que encargar del manejo de las tortugas en el área rural?** *(Who should be in charge of tortoises' management in the rural area?)*
☐El parque
☐Los propietarios
☐La junta parroquial
☐Las comunidades locales.
Cuales?____
☐No se
☐Otro____________________________

34. **Alguna vez vio tortugas en dificultad (patas arriba, enredada en alambre, heridas)?** *(Have you ever seen tortoises in trouble (*upside down*, trapped in the barbed wire, wounded)?)*
☐Patas arriba
☐Enredada en alambre
☐Herida
(Como?____)
☐Nunca he visto tortuga en dificultad
☐Otro____________________________

35. **Si ve/o si viera tortugas en dificultad (patas arriba, enredada en alambre, heridas) que hizo/que haría?** *(what did you do/what would you do when you saw/see a tortoise in trouble?)*
☐La ayudo
☐La dejo y no la toco
☐Llamo al PNG
☐Llamo a un veterinario
☐Otro____________________________

36. **Según usted las tortugas de Santa Cruz están en peligro de extinción?** *(Do you think that Santa Cruz giant tortoises are facinfig a risk of extinction?)*
☐ SI (Porque/cuáles son las amenazas para la conservación de las tortugas gigantes? Como invertiría usted?____)
☐ No
☐ No se

37. **Si fuera necesario por la conservación de las tortugas seria usted dispuesto a dejar corredores para el paso de las tortugas?** *(If it will be necessary for tortoises' conservation, would you allow giant tortoises to pass through your farm?)*
☐Ya lo hago
☐No
☐Si
☐No se
☐Otro____

38. La isla de Santa Cruz necesita inversión en temas de conservación de la naturaleza? *(Do Santa Cruz island needs more investments in conservation?)*

☐ SI (Según usted cuales son las prioridades?_________________Porque?___________________
_)

☐ No

☐ No se

Comentarios *(Comments)*
