# Supplementary material for: Emergent conservation conflicts in the Galapagos Islands: Human-giant tortoise interactions in the rural area of Santa Cruz Island
Source: PLoS One. 2018 Sep 12;13(9):e0202268. doi: 10.1371/journal.pone.0202268 (PMC6135374; doi:10.1371/journal.pone.0202268)
Supplement: S3 Table — (PDF) [file pone.0202268.s003.pdf]

**S3 Table.** Second phase: Socio-economic questionnaire. Adapted and modified from Osborn [58], Nyirenda et al. [59]

Sector:
Nombre y apellido:
Sexo:
Edad:
Nombre de la UPA:
Cargo en la UPA:
Coordinadas de GPS:
Altura del cercado por tipo de producción:

Forma No.
Numero de Teléfono

| | **Preguntas en español** | ***Questions in english*** |
|---|---|---|
| | **I. Pérdidas percibidas, incluyendo las valuaciones contingentes de disposición a pagar (DAP) y la disposición a aceptar (DAA)** | ***I. Perceived losses, including willingness to pay (WTP) and willingness to act (WTA) contingent valuation*** |
| 1 | ¿Cuál es su actividad económica primaria? | *What is your main economic activity?* |
| 2 | La UPA es primariamente: | *The land is primary for?* |
| | Ganadera, turismo, agrícola, mixta | *Cattle rearing, tourism, crop cultivation, mixed* |
| | ¿Para qué usa los productos de la upa? | *What do you use the products for?* |
| 3 | ¿La UPA bordea con el Parque? | *Does the land borders with the park?* |
| 4 | ¿Cuántas hectáreas tiene la UPA? | *How many ha does it have?* |
| 5 | ¿Entran tortugas en su UPA? | *Do tortoises enter the land?* |
| 6 | ¿Causan algún daño? | *Do tortoises cause damage?* |
| 7 | ¿Cuándo (en qué mes(es), temporada) fue la última vez que tuvo daños y a qué? | *When (months, season) was the last time you had damage and to what?* |
| 8 | ¿Para qué hubiese usado el “producto” dañado? (venta, consumo, vacas, protección) | *What would the damaged product have been used for??* |

**II. Para cercas**

1 ¿Estime los metros de cercas que necesita en sus campos para evitar daños por las tortugas?

2 Sí previamente sufrió de danos a sus cercas, cuanto fue el daño en (metros) y términos monetarios (dólares-USD) por campo?

***II. To fences***

*Estimate the meters of fences you need in your fields to avoid damage by tortoises?*

*If you previously suffered from damage to fences, how much was damage in (mts) and monetary terms (USD) per field?*

**III. Para las tierras con cultivos**

1 Estime el tamaño de sus terrenos/campos dañados:

2 De estas últimas perdidas en sus terrenos dañados por las tortugas, ¿Cuánto perdió por la invasión de las tortugas en área (m²) y términos monetarias (dólares-USD)?

3 Daños a los cultivos

¿Cuál fue el cultivo, daño, calidad del cultivo y edad/etapa de crecimiento del cultivo?

***III. For lands with crops***

*Estimate the average size (in m²) of your fields:*

*If you previously suffered loss from crop damage, how much did you lose to crop raiding in area (m²) and monetary terms (USD) per crop type?*

*Crop damages*

*What was the crop, damage, quality of the crop and age/growth stage of the crop?*

Categoría del daño

*Damage category*

1=<5%
2=6-10%
3=11-20%
4=21-50%
5=51-80%
6=>80%

| **Español** | Daño | Calidad antes del daño | | | | Edad del cultivo | | |
|---|---|---|---|---|---|---|---|---|
| Cultivo | Tipo | BUENO | MEDIO | MALO | | Plántula | Intermedio | Maduro |
| Cultivo 1 | .. | | | | | | | |
| Cultivo 2 | .. | | | | | | | |
| Cultivo 3 | .. | | | | | | | |

| | | | | | | | | |
|---|---|---|---|---|---|---|---|---|
| Cultivo 4…… | .. | | | | | | | |

| ***English*** | *Damage* | *Quality before damage* | | | | *Crop age* | | |
|---|---|---|---|---|---|---|---|---|
| *Crop* | *Type* | *GOOD* | *MEDIUM* | *POOR* | | *Seedling* | *Intermediate* | *Mature* |
| *Crop1* | .. | | | | | | | |
| *Crop2* | .. | | | | | | | |
| *Crop3* | .. | | | | | | | |
| *Crop4* | .. | | | | | | | |
| *Crop…..* | .. | | | | | | | |

---

| | | |
|---|---|---|
| 4 | ¿Cómo compensó las pérdidas? | *How did you make up for the loss?* |
| 5 | ¿Cuál es la mejor precio al cual hubiese podido vender sus cultivos? | *Which is the greatest opportunity cost for your growing crops?* |
| 6 | ¿Cuánto hubiese ganado en la última temporada si hubiese cultivado algo diferente y menos vulnerable a la invasión de las tortugas? (Existe esto?) | *How much would you have gained in the last farming season if you cultivated a different and less vulnerable crop type to invading by tortoises (does it exist?)* |
| 7 | ¿Qué medidas utiliza para que las tortugas no entren en su terreno? ¿Éstas medidas son sólo para tortugas? | *What meassures do you use to avoid tortoises entering your land? Are these measures only for tortoises?* |
| 8 | ¿Cuánto invierte para estas medidas en tiempo y dinero? | *How much do you invest in these measure both in time and money?* |
| 9 | ¿Cuánto estaría dispuesto a pagar para que las tortugas no entren en su terreno? | *How much would you be willing to pay to improve protection against crop raiding?* |

---

## IV. Cercas dañadas en el ultimo año

## *IV. Damaged fences in the last year*

| | | |
|---|---|---|
| 1 | En que mes(es) durante el último año tuvo de daños en sus cercas por las tortugas? (inferida en P7) | *Which month(s) during the last year, did you suffer from fence damage by tortoises?* |
| 2 | ¿Estime los metros de cercas que necesita en sus campos para evitar daños por las tortugas? | *Estimate the meters of fences you need in your fields to avoid damage by tortoises?* |
| 3 | Sí previamente sufrió de danos a sus cercas, cuanto fue el daño en (metros) y términos monetarios (dólares-USD) por campo? | *If you previously suffered from damage to fences, how much was damaged in(m) and monetary terms (USD) per field?* |
| **V. Alternativas y perspectivas locales** | | ***V. Local alternatives and perspectives*** |
| ¿Qué sugiere debería hacerse para evitar que las tortugas le causen daños? | | *What do you suggest should be done to alleviate or reduce crop raiding?* |
