# Supplementary figures and images for: Emergent conservation conflicts in the Galapagos Islands: Human-giant tortoise interactions in the rural area of Santa Cruz Island
Source: PLoS One. 2018 Sep 12;13(9):e0202268. doi: 10.1371/journal.pone.0202268 (PMC6135374; doi:10.1371/journal.pone.0202268)

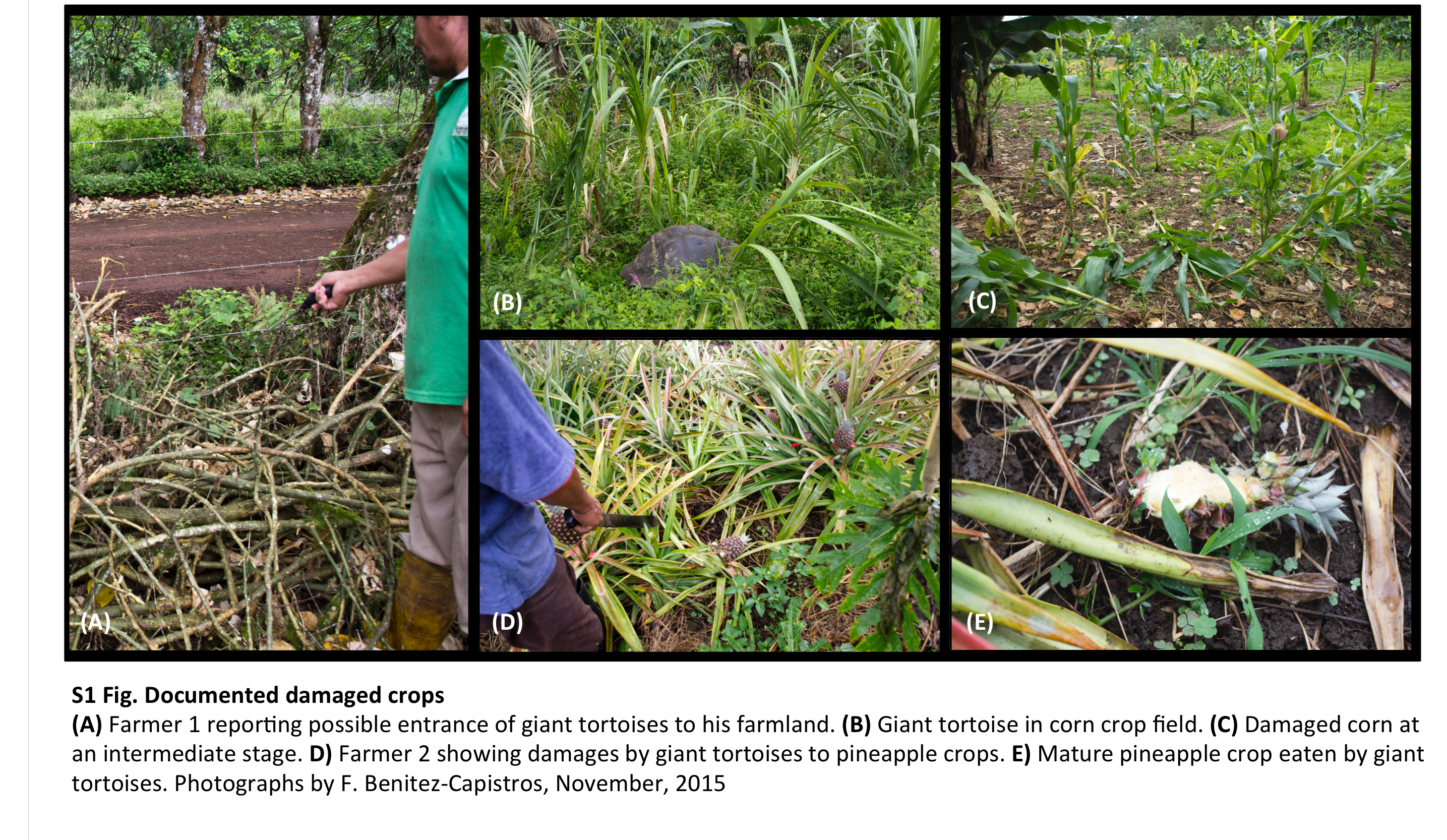

Supplement: S1 Fig — a) Farmer 1 reporting possible entrance of giant tortoises to his farmland; b) giant tortoise in corn crop field; c) damaged corn at an intermediate stage; d) farmer 2 showing damages by giant tortoises to pineapple crops; e) mature pineapple crop eaten by giant tortoises. Photographs by F. Benitez-Capistros, November, 2015. (TIFF) [file pone.0202268.s004.tiff]

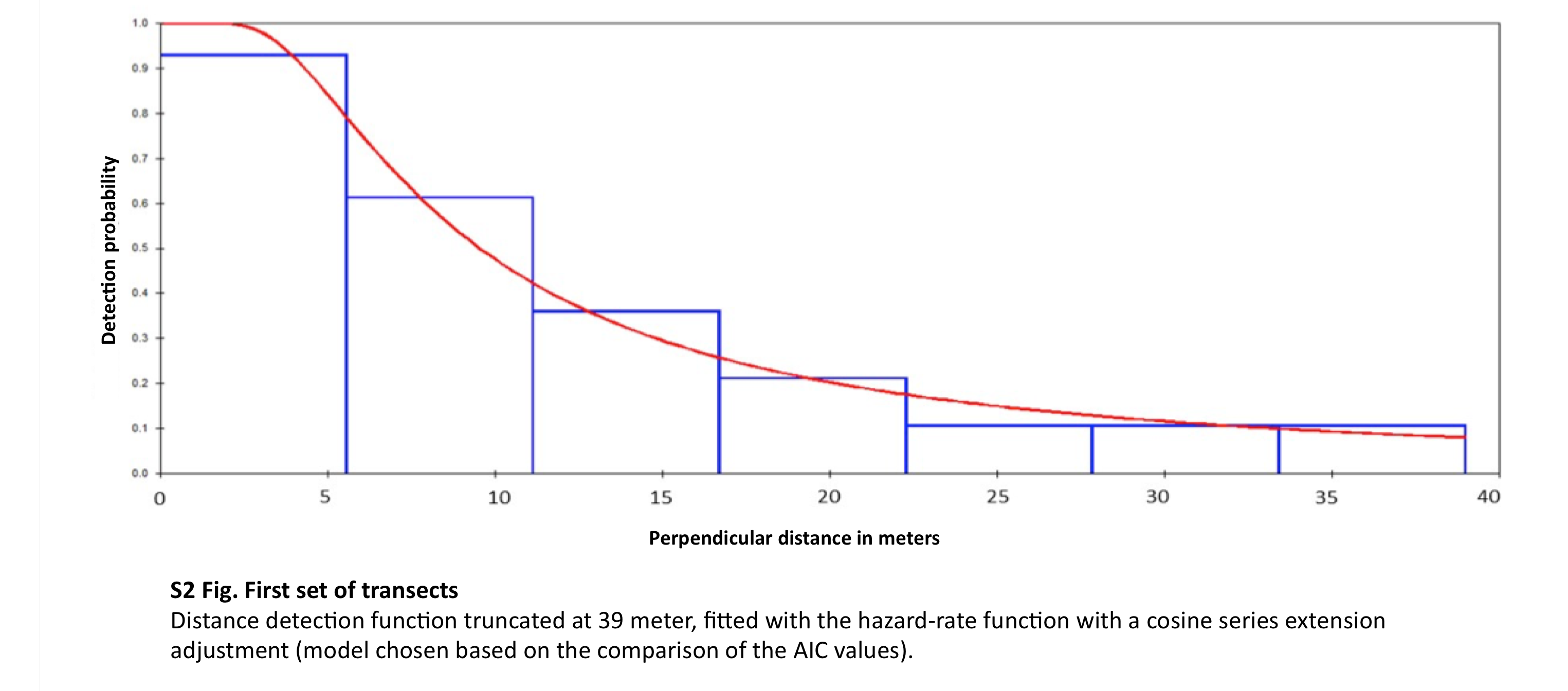

Supplement: S2 Fig — Distance detection function truncated at 39 meter, fitted with the hazard-rate function with a cosine series extension adjustment (model chosen based on the comparison of the AIC values). (TIFF) [file pone.0202268.s005.tiff]

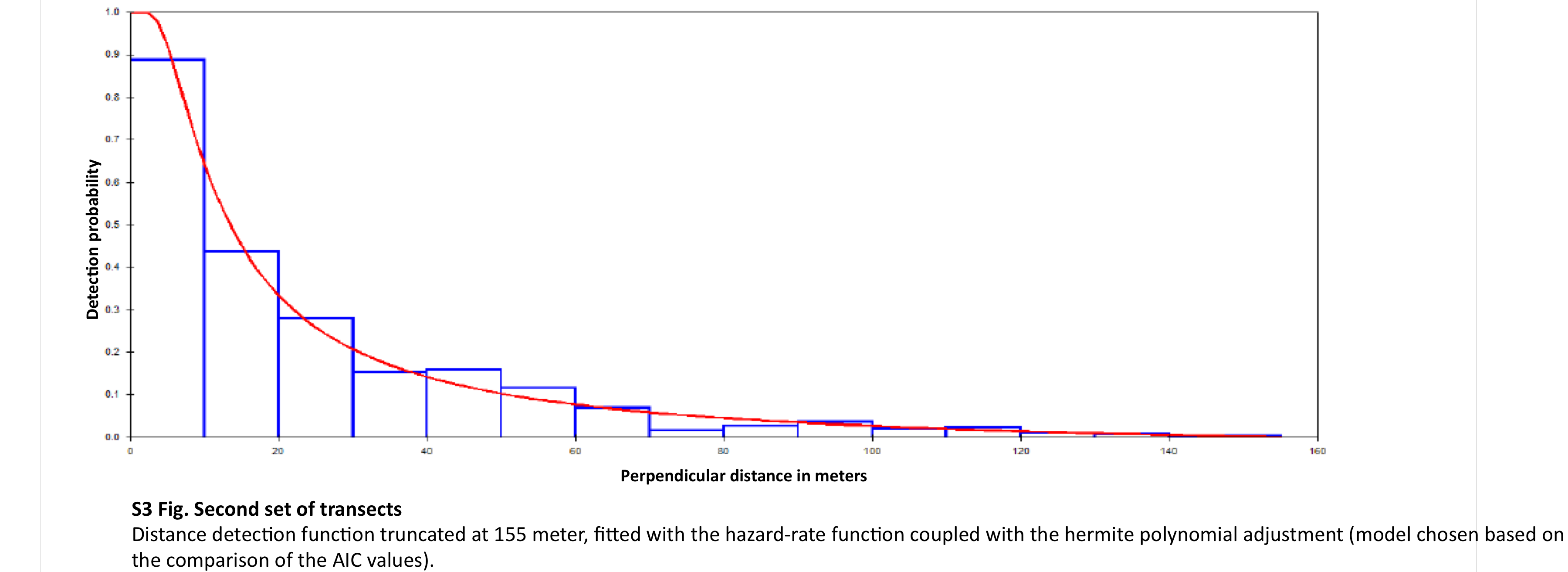

Supplement: S3 Fig — Distance detection function truncated at 155 meter, fitted with the hazard-rate function coupled with the hermite polynomial adjustment (model chosen based on the comparison of the AIC values). (TIFF) [file pone.0202268.s006.tiff]
